# Supplementary material for: Brassinosteroid signaling delimits root gravitropism via sorting of the Arabidopsis PIN2 auxin transporter
Source: Nat Commun. 2019 Dec 4;10:5516. doi: 10.1038/s41467-019-13543-1 (PMC6892858; doi:10.1038/s41467-019-13543-1)
Supplement: Supplementary file 1 — Supplementary Information [file 41467_2019_13543_MOESM1_ESM.pdf]

Supplementary Information for:

'Brassinosteroid signaling delimits root gravitropism via sorting of the Arabidopsis PIN2 auxin transporter'

Retzer et al.

SUPPLEMENTARY NOTES

Supplementary Note 1 – A model of IAA transport in the root apex

To assess effects of PIN2 asymmetry in the root apex on intracellular auxin concentration and its temporal dynamics, we simulated auxin transport in the root tip. For this purpose, we transformed our recently developed 3D model [1] into 2D (Supplementary Fig.11a). The modeled root tip comprises a multicellular structure with isolated intracellular compartments and an extracellular compartment (the cell wall), within which auxin can diffuse freely, following the framework used in previous models of auxin transport [2, 3, 4]. Auxin transport between adjacent compartments is governed by its concentration gradient and membrane permeability. There are three distinct paths for auxin to permeate the cell membrane: diffusion, influx carriers and efflux carriers. A major governing factor for auxin transport is the distribution of membrane permeabilities for auxin, which depend on localization of the carrier proteins (AUX1, PINs and others) [2, 3, 4]. Our model also contains a media compartment around the root that makes it possible to apply boundary conditions simulating experiments, in which roots are placed on top of the agar block and thus have contact with agar gel, where auxin can diffuse (see Methods).

Supplementary Note 2 – Model Geometry

Presenting the root tip as an array of rectangular cells has computational advantages and provided useful quantitative results in the past [2, 4, 5]. We draw a 2D rectangular geometry of a root tip, containing cells of the root apex: meristematic zone, elongation zone and the beginning of the differentiation zone (Supplementary Fig.11a). This schematic root apex is ~ 1200 μm in length and 160 μm in width. The outmost domain represents the surrounding growth medium (agar block). Widths of the cell files in our model correspond to those in the elongation zone, taken from sectioned confocal images [3]. The heights of the cells are also taken from confocal images. Lateral root cap cells are drawn as an additional cell file on the outer side of the meristematic zone. Cell wall thickness is set to 200 nm.

The geometry presented in Supplementary Fig.11a serves as a main model. In order to test dependence of the results of our simulations on root geometry, we transformed the main geometry, and also tested a geometry, which is based on the image of a root cross section (Supplementary Fig.11cd, Supplementary Fig.12). These model variants are described below in Supplementary Note 2.2.

Supplementary Note 3 - Parameters governing auxin transport; specifying the carrier distributions

Bulk diffusion:

Diffusion coefficients inside the cell are assumed to be equal to diffusion in water at room temperature $D_c = 600\ \mu m^2/s$ [2]; in the cell wall $D_w = 32\ \mu m^2/s$ based on published measurements [6] and in media $D_m = 60\ \mu m^2/s$ because diffusion hindrance of a small molecule in 1% agar is ~0.1 compared to water solution.

Carrier distribution:

Membrane transport of auxin has three components: 1) diffusion of protonated IAAH, 2) influx of the IAA^- anion from the cell wall into the cellular interior via influx carriers, 3) efflux of IAA^- from the cell into the cell wall domain via efflux carriers (Supplementary Fig.11a). Diffusional permeability is the same for all cell boundaries. Carrier-mediated membrane permeabilities depend on carrier density and on the specific permeability of the unit density of a carrier. Carrier densities are assigned for each cell boundary, following the carrier localization schemes presented in Simuplant software, using default settings [3]: based on the fluorescence staining of the main known auxin transporters in the root (AUX1, LAX2 and LAX3 influx transporters; PIN1, PIN2, PIN3, PIN4 and PIN7 efflux transporters), it has been assumed that either carrier is present on the membrane and its density equals 1, or is absent, with a density equaling 0 (Supplementary Fig.11a). Most cells containing influx carriers, have them localized on all sides of the cell membrane [3]. AUX1 is localized primarily in outward cell layers: lateral root cap (LRC) and epidermis. LAX2/LAX3 is localized in the stele. PIN proteins are polarized in most cells (columella cells under QC are an exception, where PINs are present on all boundaries) and most cells have a prevailing PIN-type expressed. This results in the characteristic distribution of PINs within the root [3], establishing rootward auxin flux in the stele and shootward auxin flux in the outer cell layers [4]. PIN1 is localized basally and baso-laterally in the stele. PIN2 is localized apically in the epidermis and cortex and in the opposite direction (basally) in cortical cells proximal to the QC. Additional non-polar efflux transporters (NPEs) are not yet fully characterized well, but they are suggested to exhibit less permeability [3]. For this reason, we neglected NPEs in our simulations.

Measured permeability values:

Permeability values have been measured separately for diffusion, influx and efflux carriers in a number of studies [7, 8]. Rutschow and colleagues [7] provide diffusion permeabilities for *Arabidopsis* root protoplasts ($P_{IAAH} = 0.35 \pm 0.22\ \mu m/s$), which is close to the values provided by [8] for tobacco cells. Influx permeability in AUX1-positive root protoplasts was determined [7]

as: $P_{\text{AUX1}} = 0.35 \pm 0.07\ \mu\text{m/s}$. We assume $P_{\text{LAX2,3}} = P_{\text{AUX1}}$ [3]. Permeability for PINs has been previously estimated as ~ $0.3\ \mu\text{m}/s$ [2]. One strategy to estimate PIN efflux permeabilities in epidermis cell files is based on polar auxin transport (PAT) velocities [8]. Measured auxin velocities in the *Arabidopsis* root are in a range of 0.3 to $3\ \mu\text{m}/s$ [1]. In our model, we set the efflux permeability of PIN2 value, such that the shootward auxin flux in the epidermis equals $1\ \mu m/s$. Such velocity is produced by $P_{PIN2} = 0.5 \pm 0.2\ \mu\text{m/s}$. This is close to the value estimated by Rutschow et al. [7]. We assume that all PIN-containing cells have the same P_{PIN}, disregarding different PIN types and their overlapping expression in certain cell files. Thus, $P_{\text{PIN1}} = P_{\text{PIN3/PIN7}} = P_{\text{PIN2}} = 0.5\ \mu\text{m/s}$. This assumption is rough, however, as we aim to calculate $[\text{IAA}]_{\text{cell}}$ mainly in the epidermis, we choose P_{AUX1} and P_{PIN2} as the critical values for this model.

To simulate asymmetry of PIN2 at the upper and lower side of the root apex we grouped all shootward-PIN2-containing membranes, as depicted in the schemes (Fig. 8a-d), on the upper side and on the lower side. Changes in $P_{\text{PIN2}}^{\text{upper side}}$ and $P_{\text{PIN2}}^{\text{lower side}}$ were applied for all membranes in one group.

Other assumptions:

(i) Saturation of the auxin transporters is not considered, because concentrations tested in the model are below saturation level: $K_{\text{D(AUX1)}} \cong 2\mu\text{M}$, $K_{\text{D(PINs)}} \cong 6\ \mu\text{M}$ [9, 10], whereas calculated $[\text{IAA}]_{\text{cell}} < 1.5\ \mu\text{M}$ (to account for IAA concentration and not only relative concentration we set the boundary condition $\text{IAA}_{\text{shoot}}$ = 40 nM, which produces average steady state $[\text{IAA}]_{\text{cell}}$ of ~ 100 nM, consistent with published experimental data on average IAA content in the root tip [12] (for $\text{IAA}_{\text{shoot}}$ = 40 nM intracellular $[\text{IAA}]_{\text{cell}} < 1.5\ \mu\text{M}$ for all our simulations).

(ii) Auxin synthesis and degradation are neglected in the model, since no clearly defined values are available for the analyzed cell files. Nevertheless, Kramer and Ackelsberg estimated that synthesis contributes less than 1% of the auxin amount present in fast transporting tissues such as roots [11]. Auxin degradation is a slow process compared to transport [3, 11]. As we have an infinite pool of auxin (coming from the shoot), we considered degradation as negligible.

For the complete list of the parameters used in the model, see Supplementary Table 1.

Supplementary Note 4 - Governing equations: auxin diffusion within compartments and membrane auxin fluxes

Auxin concentration is calculated by solving a system of Partial Differential Equations (PDEs) that describes how the auxin concentration $\text{IAA}(x, t)$ within each compartment evolves due to

bulk auxin diffusion (Fick's law) and the mass conservation law:

$$\frac{\partial[IAA]_{c/w/m}}{\partial t} = D_{c/w/m} \cdot \Delta[IAA]_{c/w/m}. \quad (1)$$

Normal diffusive auxin flux at the boundaries of compartments equals auxin flux across membrane between compartments:

$$J_{\text{boundary}} = -D_{c/w/m} \cdot \nabla[IAA]_{c/w/m} = J_{\text{wall}\to\text{cell}} \quad (2)$$

(=molecules passed through membrane per unit time per unit area), which is governed by membrane permeabilities:

$$J_{\text{wall}\to\text{cell}} = J_{\text{diff}} + J_{\text{influx}} + J_{\text{efflux}}\ , \quad (3)$$

$$J_{\text{diff}} = P_{\text{IAAH}}(\text{A}_1 \cdot [\text{IAA}]_{\text{wall}} - \text{B}_1 \cdot [\text{IAA}]_{\text{cell}}) \quad (4)$$

$$J_{\text{influx}} = P_{\text{AUX1 or LAX2,3}}(\text{A}_2 \cdot [\text{IAA}]_{\text{wall}} - \text{B}_2 \cdot [\text{IAA}]_{\text{cell}}) \quad (5)$$

$$J_{\text{efflux}} = P_{\text{PIN}}(\text{A}_3 \cdot [\text{IAA}]_{\text{wall}} - \text{B}_3 \cdot [\text{IAA}]_{\text{cell}}), \quad (6)$$

where

$$A_1 = \frac{1}{1+10^{pH_{wall}-pK}},\quad B_1 = \frac{1}{1+10^{pH_{cell}-pK}}\ ,$$

$$A_2 = q(\tilde{\phi})(1-A_1),\ B_2 = q(-\tilde{\phi})(1-B_1),$$

$$A_3 = q(-\tilde{\phi})(1-A_1),\ B_3 = q(\tilde{\phi})(1-B_1),$$

$$q(x) = x/(e^x - 1),\ \tilde{\phi} \equiv F_{\text{D}}V_{\text{m}}/RT \quad (7)$$

which depend on acidity of the wall pH_{wall}, cytoplasmic pH_{cell}, the logarithmic dissociation constant of auxin pK and the membrane potential V_{m}. F_{D} is the Faraday constant, R is the gas constant and T is the temperature. Derivation of A_i, B_i is provided in detail in [2] and [3]. For $pH_{wall} = 5.3$, $pH_{cell} = 7.2, V_{\text{m}} = -120mV$, typical for the root, these constants have the following values: $A_1 = 0.24, B_1 = 0.004$, $A_2 = 3.57, B_2 = 0.045, A_3 = 0.034, B_3 = 4.68$, giving the following equations for fluxes from Supplementary Eq, 4,5,6:

$$J_{\text{diff}} = P_{\text{IAAH}}(0.24 \cdot [\text{IAA}]_{\text{wall}} - 0.004 \cdot [\text{IAA}]_{\text{cell}}) \quad (8)$$

$$J_{\text{influx}} = P_{A\text{UX1 or LAX2,3}}(3.57 \cdot [\text{IAA}]_{\text{wall}} - 0.045 \cdot [\text{IAA}]_{\text{cell}}) \quad (9)$$

$$J_{\text{efflux}} = P_{\text{PIN}}(0.034 \cdot [\text{IAA}]_{\text{wall}} - 4.68 \cdot [\text{IAA}]_{\text{cell}}), \quad (10)$$

Supplementary Note 5 - External boundary conditions (Supplementary Fig.11a,e).

At the upper boundary of the stele, the IAA concentration is set to constant $[\text{IAA}]_{\text{shoot}}$ = 40nM that corresponds to the supply of auxin from the shoot. At the upper boundary of the epidermis the auxin sink is set by concentration condition: $[\text{IAA}]_{\text{epi}}$ = 0. The external vertical boundary of the media compartment is characterized by periodic boundary conditions. The remains of the external boundaries have a 'no flux' boundary condition. IAA concentration is presented in all figures in non-dimensional units relative to $[\text{IAA}]_{\text{shoot}}$: $[\text{IAA}]_{\text{cell}}$/ $[\text{IAA}]_{\text{shoot}}$.

Supplementary Note 6 - Initial conditions

We considered two cases:

Case 1 No gravistimulation: First, we calculate a steady-state solution from initial $[IAA]_{cell} = 0$, $[IAA]_{wall} = 0$ and $P_{\mathrm{PIN3/PIN7}} = P_{\mathrm{PIN2}}^{\mathrm{upper\ side}} = P_{\mathrm{PIN2}}^{\mathrm{lower\ side}} = P_{\mathrm{PIN1}} = P_{\mathrm{PIN}}^{\mathrm{initial}}$ (all PIN permeabilities are equal; Fig. 8a; Equation 1). The resulting [IAA] distribution corresponds to a downward-oriented root. This distribution serves as an initial condition for further numerical analysis of IAA dynamics after the onset of the PIN2 asymmetry (for Fig. 8b,e).

Case 2 Gravistimulation*:* When simulating gravistimulated roots, the initial conditions are the same as in case of no stimulation, except for columella cells containing $P_{\mathrm{PIN3/PIN7}}$ (see Fig. 8c for a scheme). Permeability on the membrane of the lower side of these cells is set higher: $P_{\mathrm{PIN3/PIN7}}^{\mathrm{lower\ side}} = k_{\mathrm{PIN3gravi}} \cdot P_{PIN3/PIN7}^{0} + k_{\mathrm{PIN3all}} \cdot P_{PIN3/PIN7}^{0}$, with $k_{\mathrm{PIN3gravi}}>1$ and $k_{\mathrm{PIN3all}} < 1$. And permeabilities on all other membranes of these cells are set lower: $P_{PIN3/PIN7}^{all\ sides} = k_{\mathrm{PIN3all}} \cdot P_{PIN3/PIN7}^{0}$, with $k_{\mathrm{PIN3all}} < 1$. Sum of permeabilities for each cell is kept the same as in the "No gravistimulation" case. The corresponding ratio of auxin fluxes to the lower vs to the upper side of the root is calculated from the average auxin fluxes through the left-most membranes of columella cells and through the right-most membranes of columella cells, $\frac{\mathrm{IAA\ flux}^{\mathrm{lower}}}{\mathrm{IAA\ flux}^{\mathrm{upper}}}$. We use this ratio further as a measure of "gravistimulation strength". Parameter values correspond to $\frac{\mathrm{IAA\ flux}^{\mathrm{lower}}}{\mathrm{IAA\ flux}^{\mathrm{upper}}}$ as follows:

Auxin flux ratio	$\frac{\mathrm{IAA\ flux}^{\mathrm{lower}}}{\mathrm{IAA\ flux}^{\mathrm{upper}}}$	$\frac{95\%}{5\%}$	$\frac{85\%}{15\%}$	$\frac{70\%}{30\%}$	$\frac{65\%}{35\%}$	$\frac{60\%}{40\%}$	$\frac{50\%}{50\%}$
Lower side permeability factor	$k_{\mathrm{PIN3gravi}}$	4	2.4	1.6	1.36	1.12	0
All sides permeability factor	k_{PIN3all}	0	0.4	0.6	0.66	0.72	1

The resulting steady-state distribution of IAA serves as an initial condition for further numerical analysis of IAA distribution after onset of the PIN2 asymmetry in the case of gravistimulation (Fig. 8c).

Supplementary Note 7 - PIN2 asymmetry simulation (Main model)

PIN2 distribution in root tip cells is simplified in the model, by assuming that PIN2-mediated permeability of all PIN2-containing membranes on each side of the root is the same (at the lower and at the upper side; Fig. 8a,b schemes, blue bars). Accordingly, we introduced two variables for auxin permeabilities: $P_{\mathrm{PIN2}}^{\mathrm{lower\ side}}$ and $P_{\mathrm{PIN2}}^{\mathrm{upper\ side}}$. To restrict our investigation to shootward auxin transport, we varied permeabilities only at apical PIN2-containing membranes (Supplementary Fig.11a; Supplementary Table 1).

We assumed that PIN2 effects on auxin membrane permeability are proportional to PIN2 abundance and, accordingly, the ratio of permeabilities is proportional to the PIN2:Venus signal ratio, as presented in Fig. 6e (Equation 2): $\frac{P_{\mathrm{PIN2}}^{\mathrm{upper\ side}}}{P_{\mathrm{PIN2}}^{\mathrm{lower\ side}}} = \frac{PIN2{:}Venus\ signal\ upper\ side}{PIN2{:}Venus\ \ signal\ lower\ side} = k$. We use this ratio, denoted as a parameter k in the model, to define permeabilities on the sides as functions of the initial permeability (Equations 3 and 4):

$$P_{\mathrm{PIN2}}^{\mathrm{upper\ side}} = P_{PIN2}^{initial} \cdot \sqrt{k} \quad (11)$$

$$P_{\mathrm{PIN2}}^{\mathrm{lower\ side}} = \frac{P_{PIN2}^{initial}}{\sqrt{k}}. \quad (12)$$

Thus, in this variant of the model we assume that both permeabilities, on the upper and on the lower side depend on k: for example, when PIN2 becomes asymmetric with $k < 1$, $P_{\mathrm{PIN2}}^{\mathrm{upper\ side}}$ decreases and $P_{\mathrm{PIN2}}^{\mathrm{lower\ side}}$ increases. This is an unverified assumption, because experimental data in our present study provides only values of the PIN2 signal ratio (k). However, previous studies [12] presented some evidence that indeed PIN2 abundance is affected on both sides of the root apex. Supplementary Eq. 11 and 12 are applied to all PIN2-containing membranes in groups at the "upper side" and "lower side", as depicted in Fig. 8a.

Supplementary Note 8 - Numerical procedures

PDEs (Supplementary Eq. 1 in compartments and Supplementary Eq. 2, 3, 8, 9,10 on boundaries) are solved using finite element software Comsol Multiphysics 5.3a: domains of the 2D geometry are discretized by constructing a rectangular mesh inside the cells (element size ~0.5 um – 1 um, comprising ~100 -1000 elements per cell) and triangular-element mesh in the apoplast (0.2 um size) and media (1 um – 10 um size). Variables are defined in vertices and computed over time. Stationary solver was used to calculate the steady-state IAA concentrations and average of those concentrations over 5 cells in the EZ, that were plotted on the graphs (Fig. 8gh, Supplementary Fig. 12c, Supplementary Fig.13d, Supplementary Fig.14e, Supplementary Fig.15ab, Supplementary Fig.16ad, Supplementary Fig.17). For the calculation of the temporal dynamics of IAA after the onset of PIN2 asymmetry, the steady state solution was used as an initial IAA concentration (Fig. 8e,f). Time-dependent solver was

then used to calculate IAA concentration at each time step, which were determined by the solver.

Supplementary Note 9 – Results of simulation

Calculated steady state $[IAA]_{cell}$ for the range of $k = 0.05 - 1.4$ is used to assess an average $[IAA]_{cell}$ within the 5 first cells of the root elongation zone on the upper and on the lower side of the root (blue and green dots on the scheme in Fig. 8a). The ratio of these average concentrations, normalized by $[IAA]_{shoot}$ is plotted in Fig. 8g: $\frac{\mathrm{IAA}^{\mathrm{lower}}}{\mathrm{IAA}^{\mathrm{upper}}}(k)$.

Results of the main model are presented in Fig. 8 and described in the main text. Next, we estimated the sensitivity of our conclusions derived from the main model, to variations in model parameters, including root shape, type and location of boundary conditions, PIN2 localization, initial PIN2 permeability as well as AUX1 permeability (Supplementary Fig.11-17).

Supplementary Note 10 - Effect of the root geometry

We tested several root geometries to assess the sensitivity of the accumulation ratio $\frac{\mathrm{IAA}^{\mathrm{lower}}}{\mathrm{IAA}^{\mathrm{upper}}}(k)$ to the relative position and cell shape. For all geometries, we calculated $\frac{\mathrm{IAA}^{\mathrm{lower}}}{\mathrm{IAA}^{\mathrm{upper}}}(k)$ for "Case 1, no gravistimulation" and "Case 2, gravistimulation". In gravistimulation case we seek to compare resulting $\frac{\mathrm{IAA}^{\mathrm{lower}}}{\mathrm{IAA}^{\mathrm{upper}}}$ for the same strength of gravistimulation, which is defined by the asymmetry of PIN3/7 permeability on the lower side of columella cells. Thus, we compare all geometries gravistimulated with the same strength, $\frac{\mathrm{IAA\ flux}^{\mathrm{lower}}}{\mathrm{IAA\ flux}^{\mathrm{upper}}} \sim = \frac{70\%}{30\%}$.

Two general conclusions from this investigation are as follows:

First, the main qualitative result of the model remains unchanged in all variants of the model. Namely, the relative auxin concentration inside the cell is reversely proportional to the PIN2 permeability given that other transporters do not differentially change on upper/lower side of the root. As $\frac{\mathrm{IAA}^{\mathrm{lower}}}{\mathrm{IAA}^{\mathrm{upper}}}$ is calculated always for the symmetrically situated cells having similar geometries and proportions of transporters, differential PIN2 permeability in these cells is the only parameter influencing $\frac{\mathrm{IAA}^{\mathrm{lower}}}{\mathrm{IAA}^{\mathrm{upper}}}$.

Second, in all the models we have observed a slight change in the slope $\frac{\mathrm{IAA}^{\mathrm{lower}}}{\mathrm{IAA}^{\mathrm{upper}}}(k)$ in case of gravistimulation (Supplementary Fig.11g, Supplementary Fig.14e). This result shows that the initial $\frac{\mathrm{IAA}^{\mathrm{lower}}}{\mathrm{IAA}^{\mathrm{upper}}}$ caused by PIN3/7 asymmetry depends not only on the degree of PIN3/7 asymmetry (and corresponding gravistimulation strength), but also on cell shapes. This is due to the fact that relative lengths of the sides of the cell defines the proportion of influx and efflux

carriers and thus influences differential auxin fluxes.

Resulting graphs for $\frac{\mathrm{IAA}^{\mathrm{lower}}}{\mathrm{IAA}^{\mathrm{upper}}}(k)$ in all tested models are shown on Supplementary Fig.12c, Supplementary Fig.13d, Supplementary Fig.14e.

1) Elongated geometry (Supplementary Fig.11bcd).

Elongated geometry has an extended length of the differentiation zone (DZ) up to 3500 um (Supplementary Fig.11b). Two variants of this model were investigated:

1.1 Influx and efflux carrier localization in all added cells are equal to those of the upper cell layer of the main model (Supplementary Fig.11c, results Supplementary Fig.12c).

1.2 Cells in DZ above 1200 um do not contain PIN2 and AUX1, cells in the stele above 1200 um contain basal PIN1 (Supplementary Fig.11d, results Supplementary Fig.12c). This setup aims to elucidate the effect of gradually declining PIN2 and AUX1 membrane abundance in the shootward direction [13]. Complete absence of PIN2 and AUX1 is thus an oversimplification and leads to increased accumulation of auxin in those epidermis cells (Supplementary Fig.11d, cells of DZ above 1200 um). However, in this setup the effect of PIN2 asymmetry remains the same as in the main model, independent of auxin concentrations in downstream cells (Supplementary Fig.12c). In case of gravistimulation, effects of PIN2 asymmetry are also similar to the main model (graph presented in Supplementary Fig.12c).

2) Realistic image-based geometry (Supplementary Fig.12a).

To evaluate the influence of the squarish cell geometry on auxin distribution, we constructed a realistic root geometry using a published 2D root image, modified from the Simuplant model (Fig. 1e from [3]) to allow comparison with Simuplant results. Localization of PINs and AUX1 carriers are taken from the Simuplant model (from Fig. 1e and Fig. 2a [3].) The effect of the PIN2 asymmetry $\frac{P_{\mathrm{PIN2}}^{\mathrm{upper\ side}}}{P_{\mathrm{PIN2}}^{\mathrm{lower\ side}}}$ on $\frac{\mathrm{IAA}^{\mathrm{lower}}}{\mathrm{IAA}^{\mathrm{upper}}}$ remains the same as in the main model (Supplementary Fig.12c). However, in the case of gravistimulation, similar gravistimulation strength leads to lower auxin ratio $\frac{\mathrm{IAA}^{\mathrm{lower}}}{\mathrm{IAA}^{\mathrm{upper}}}$. This is due to very short geometry and influence of the sink boundary conditions close to the EZ cells.

3) eBL-treated root geometry (Supplementary Fig.12b)

e-BL-treated roots have more elongated cell shapes in meristem, which consists of fewer cells, and overall thickness of the root is smaller. We simulated these morphological changes in our squarish geometry and found only a slight enhancement of the initial $\frac{\mathrm{IAA}^{\mathrm{lower}}}{\mathrm{IAA}^{\mathrm{upper}}}$ in case of

gravistimulation, but the major outcome of the model, namely the positive slope of the $\frac{\mathrm{IAA}^{\mathrm{lower}}}{\mathrm{IAA}^{\mathrm{upper}}}(k)$ function, remains unchanged (Supplementary Fig.11e,g).

4) Geometry with wide lateral root cap cells (for better visualization).

LRC cells width is defined in the models (main, elongated and eBL-treated geometries) based on thickness of LRC cells in real roots (width~ 5um). We tested how auxin concentrations in EZ will depend on 2x times increase in width of LRC, and found that it leads only to slight enhancement of the initial $\frac{\mathrm{IAA}^{\mathrm{lower}}}{\mathrm{IAA}^{\mathrm{upper}}}$ in case of gravistimulation, but the positive slope of the $\frac{\mathrm{IAA}^{\mathrm{lower}}}{\mathrm{IAA}^{\mathrm{upper}}}(k)$ function, remains unchanged. Further, we use these geometries in our figures to make auxin concentrations in the LRC cells more visible (Supplementary Fig.12c).

5) Model of one cell in the media (Supplementary Fig.13a): only one cell on either side of the root is left to simulate a single cell in the media. In all other cells the membrane is eliminated and cell interiors are replaced by media. Source and sink of auxin remain in the same place as in the main model. The purpose of this minimalistic model is to calculate auxin ratio in case there is no influence of neighboring cell on the intracellular concentration, and intracellular concentration depends solely on permeabilities of the membranes of a given cell. Resulting $\frac{\mathrm{IAA}^{\mathrm{lower}}}{\mathrm{IAA}^{\mathrm{upper}}}(k)$ is presented in Supplementary Fig.13d, and does not significantly differ from the resulting $\frac{\mathrm{IAA}^{\mathrm{lower}}}{\mathrm{IAA}^{\mathrm{upper}}}(k)$ of the main model.

6) Model of isolated column of 5 cells in media: only those 5 cells of the elongation zone, in which an average auxin concentration is measured (see scheme in Fig. 8a, blue and green dots), are left in the model. In all other cells, the membrane is eliminated and the interior is replaced by media. Two types of source/sink conditions are investigated:

6.1 With "far" boundary conditions (Supplementary Fig.13b), where source and sink of auxin remain in the same place as in the main model.

6.2. With "close" boundary conditions: source and sink of auxin are situated immediately above the uppermost cell (Supplementary Fig.13c).

These examples demonstrate that the distance to the sink and source boundary influences absolute auxin concentrations, but does not influence the auxin ratio $\frac{\mathrm{IAA}^{\mathrm{lower}}}{\mathrm{IAA}^{\mathrm{upper}}}$. Because IAA concentrations are inversely proportional to PIN2 permeabilities in this model, in the same way as in the main model, we conclude that outcome of the model is independent of boundary conditions and auxin accumulation in the tip of the root. Resulting $\frac{\mathrm{IAA}^{\mathrm{lower}}}{\mathrm{IAA}^{\mathrm{upper}}}(k)$

functions are presented in Supplementary Fig.13d.

Supplementary Note 11 - Effect of boundary conditions

We further have tested the sensitivity of auxin concentration ratios to changes in external boundary conditions in the main model (depicted on Supplementary Fig.11e).

1) No-flux condition around the root surface ("close BC") leads to higher auxin concentrations, and columella-induced gravistimulation has a stronger effect on $\frac{\mathrm{IAA}^{\mathrm{lower}}}{\mathrm{IAA}^{\mathrm{upper}}}$, compared to the "Main BC", meaning that less input by gravistimulation is required to achieve the same $\frac{\mathrm{IAA}^{\mathrm{lower}}}{\mathrm{IAA}^{\mathrm{upper}}}$ as under default boundary conditions.

2) Accordingly, "far BC" lead to lower auxin accumulation (higher auxin loss) and columella-induced gravistimulation has a weaker effect on $\frac{\mathrm{IAA}^{\mathrm{lower}}}{\mathrm{IAA}^{\mathrm{upper}}}$.

3) The distance from the root tip to the "source" and "sink" boundaries ('shootward end') also influences absolute concentrations of auxin, but has no effect on auxin ratios induced by PIN2 asymmetry (see Supplementary Note 2.2.; Supplementary Fig.11c,d for elongated model results, and sections "Model of one cell in media" and "Model of isolated column of 5 cells", for explanation; Supplementary Fig.13).

Supplementary Note 12 - Effect of PIN2 localization

In the main model we investigate PIN2 asymmetry effects by varying permeabilities of apically localized PIN2 (in epidermis and cortex). In this section we tested for an effect of PIN2 asymmetry additionally arising on the lateral side of epidermis and cortex in the elongation zone and in the cortex of the meristematic zone (where PIN2 is localized basally). The results are presented in Supplementary Fig.14:

1) PIN2 localized on the lateral sides of epidermis and cortex cells, effectively relocates auxin back into the PIN1-containing cells and prevents auxin loss to the sink. Under these conditions, we observed an increased effect of gravistimulation on $\frac{\mathrm{IAA}^{\mathrm{lower}}}{\mathrm{IAA}^{\mathrm{upper}}}$ (Supplementary Fig.14a,e): for the same gravistimulation strength $\frac{\mathrm{IAA\ flux}^{\mathrm{lower}}}{\mathrm{IAA\ flux}^{\mathrm{upper}}} = \frac{70\%}{30\%}$,the resulting auxin ratio $\frac{\mathrm{IAA}^{\mathrm{lower}}}{\mathrm{IAA}^{\mathrm{upper}}}$ is higher, showing that lateral PIN2 might enhance effect of PIN3/PIN7 asymmetry on auxin ratio by returning auxin into for further transport towards to root tip. However, effects of PIN2 asymmetry ($\frac{P_{\mathrm{PIN2}}^{\mathrm{upper\ side}}}{P_{\mathrm{PIN2}}^{\mathrm{lower\ side}}}$) on $\frac{\mathrm{IAA}^{\mathrm{lower}}}{\mathrm{IAA}^{\mathrm{upper}}}$ are as observed in the main model (Supplementary Fig.14e).

2) Cortical PIN2 in the meristem. In the main model we account for the cortical PIN2 in the meristem (Supplementary Fig.11a), but assume PIN2 permeability unchanged during

gravitropic response and no PIN2 asymmetry was established in those cells ($P_{\mathrm{PIN2cortex}}^{\mathrm{upper;lower}} = P_{\mathrm{PIN2}}^{\mathrm{initial}}$). To test for possible effects, we introduced

$$P_{\mathrm{PIN2cortex}}^{\mathrm{upper\ side}} = P_{\mathrm{PIN2}}^{\mathrm{initial}} \cdot \sqrt{k} \tag{13}$$

$$P_{\mathrm{PIN2cortex}}^{\mathrm{lower\ side}} = \frac{P_{\mathrm{PIN2}}^{\mathrm{initial}}}{\sqrt{k}} \tag{14}$$

(analogously and additionally to Supplementary Eq. 11 & 12; see Supplementary Fig.14b). Auxin concentration in the “no stimulus” and “gravistimulation” scenarios, appear to be similar to the main model (Fig. 8c,d vs. Supplementary Fig.14c); (except for the LRC cells, in which highest auxin concentration appears to be not in the last LRC cell in a row). Accordingly, dependence of the auxin ratio on PIN2 asymmetry $\frac{\mathrm{IAA}^{\mathrm{lower}}}{\mathrm{IAA}^{\mathrm{upper}}}(k)$ coincide completely with the one of the main model (Supplementary Fig.14e).

Next, we completely eliminated PIN2 in these cortical cells. This results in auxin accumulation in these cells (Supplementary Fig.14d). However, calculations of $\frac{\mathrm{IAA}^{\mathrm{lower}}}{\mathrm{IAA}^{\mathrm{upper}}}$ gives results very close to those calculated for the main model (Supplementary Fig.14e). The graph describing $\frac{\mathrm{IAA}^{\mathrm{lower}}}{\mathrm{IAA}^{\mathrm{upper}}}(k)$ under gravistimulation differs only slightly from the main model (Supplementary Fig.14e). This result indicates that basal PIN2 in root cortex cells, if anything, slightly enhances the auxin gradient established by PIN3/7 asymmetry.

Supplementary Note 13 - Effect of $P_{\mathrm{PIN2}}^{\mathrm{initial}}$ values on $\frac{\mathrm{IAA}^{\mathrm{lower}}}{\mathrm{IAA}^{\mathrm{upper}}}$ in presence of PIN2 asymmetry

The effect of $P_{\mathrm{PIN2}}^{\mathrm{initial}}$ on $\frac{\mathrm{IAA}^{\mathrm{lower}}}{\mathrm{IAA}^{\mathrm{upper}}}$ in case of no PIN2 asymmetry is shown in Fig. 8g. The increase in PIN2 permeability required for the $\frac{\mathrm{IAA}^{\mathrm{lower}}}{\mathrm{IAA}^{\mathrm{upper}}}$ shift, observed when comparing control and eBL-treated values, ranges from 2-fold to 100-fold, depending on gravistimulation strength. These values were consistently higher, than the experimentally observed effects of eBL treatment on PIN2:Venus abundance (Supplementary Fig.15c). We extended Fig. 8g by taking PIN2 asymmetry in control roots into account and found that its effect contributes substantially to the shift of $\frac{\mathrm{IAA}^{\mathrm{lower}}}{\mathrm{IAA}^{\mathrm{upper}}}$ in the entire physiologically relevant range of PIN2 permeabilities: $0.1 \leq P_{\mathrm{PIN2}}^{\mathrm{initial}} \leq 10$ (Supplementary Fig.15a,b). In this case, required increase in $P_{\mathrm{PIN2}}^{\mathrm{initial}}$ is within the experimentally observed range (~1.5-fold at most). For extreme values of $P_{\mathrm{PIN2}}^{\mathrm{initial}}$, the effect of PIN2 asymmetry diminishes (see next section).

Supplementary Note 14 - Effect of $\mathbf{P}^{\mathbf{initial}}_{\mathbf{PIN2}}$ values on the slope of the $\frac{\mathrm{IAA}^{\mathrm{lower}}}{\mathrm{IAA}^{\mathrm{upper}}}(\mathrm{k})$ curve

We tested the sensitivity of the accumulation ratio $\frac{\mathrm{IAA}^{\mathrm{lower}}}{\mathrm{IAA}^{\mathrm{upper}}}(k)$ in dependence of variations in initial $P^{\mathrm{initial}}_{\mathrm{PIN2}}$ permeability values, whilst permeabilities of other transporters remained unchanged. Initial conditions for the 'No gravistimulation' case were used. Results are presented in Supplementary Fig.16a. The slope of the curve $\frac{\mathrm{IAA}^{\mathrm{lower}}}{\mathrm{IAA}^{\mathrm{upper}}}(k)$ is maximal if $\bar{P}^{\mathrm{initial}}_{\mathrm{PIN2}} \approx 1$, normalized by default value $P^{0}_{\mathrm{PIN2}} = 0.5\ \mathrm{\mu m/s}$. Curves for $\bar{P}^{\mathrm{initial}}_{\mathrm{PIN2}} = 0.5, 1, 2$ coincide (Supplementary Fig.16a, dark blue, red and blue curves)). In case of $\bar{P}^{\mathrm{initial}}_{\mathrm{PIN2}} \gg 1$, or $\bar{P}^{\mathrm{initial}}_{\mathrm{PIN2}} \ll 1$, PIN2 asymmetry does not induce asymmetric distribution of auxin (Supplementary Fig.16a, yellow curves) as $\frac{\mathrm{IAA}^{\mathrm{lower}}}{\mathrm{IAA}^{\mathrm{upper}}}(k)$ is almost horizontal. IAA steady-state levels for $\bar{P}^{\mathrm{initial}}_{\mathrm{PIN2}} = 0.01$, (which serves as a model for *pin2* mutant, *eir1-4*) show a ~10 times higher [IAA] in epidermal cells compared to $\bar{P}^{\mathrm{initial}}_{\mathrm{PIN2}} \approx 1$ (Supplementary Fig.16b) and $\bar{P}^{\mathrm{initial}}_{\mathrm{PIN2}} = 100$ shows a ~10 times lower [IAA] in epidermal cells (Supplementary Fig.16c). In both cases, PIN2 asymmetry does not substantially change [IAA] distribution (as shown on Supplementary Fig.16b,c for $k = 0.5$).

Supplementary Note 15 - Effect of PIN2 asymmetry built by PIN2 depletion from the upper side only

As reliable experimental data provides only the ratio of PIN2 abundance on upper and lower sides of the root apex, it is important to test, how results of the main model (Supplementary Eq.11&12) are affected by absolute values of $P^{\mathrm{upper\ side}}_{\mathrm{PIN2}}$ and $P^{\mathrm{lower\ side}}_{\mathrm{PIN2}}$. In previous sections, we have shown that the initial permeability $P^{\mathrm{initial}}_{\mathrm{PIN2}}$ value is influencing the effect of PIN2 asymmetry on auxin distribution. However, an absolute $P^{\mathrm{initial}}_{\mathrm{PIN2}}$ value becomes relevant for [IAA] distribution only when changed by at least 10 fold, which has not been observed during PIN asymmetry establishment. To further investigate the effect of absolute permeability values, we tested the following governing relations:

$$P^{\mathrm{upper\ side}}_{\mathrm{PIN2}} = P^{\mathrm{initial}}_{\mathrm{PIN2}} \cdot k \quad (15)$$

$$P^{\mathrm{lower\ side}}_{\mathrm{PIN2}} = P^{\mathrm{initial}}_{\mathrm{PIN2}}. \quad (16)$$

where again $k = \frac{PIN2{:}Venus\ signal\ upper\ side}{PIN2{:}Venus\ signal\ lower\ side} = \frac{P^{\mathrm{upper\ side}}_{\mathrm{PIN2}}}{P^{\mathrm{lower\ side}}_{\mathrm{PIN2}}}$, but, different from Supplementary Eq.11&12, PIN2 permeability changes only at the upper side of the gravistimulated root. Initial conditions for 'No gravistimulation' case were used. Surprisingly, the resulting curve $\frac{\mathrm{IAA}^{\mathrm{lower}}}{\mathrm{IAA}^{\mathrm{upper}}}(k)$ almost coincides with the result of the main model (Supplementary Fig.16d). Discrepancies appear only for very low values: $k < 0.2$. This result argues that the ratio

$\frac{P_{\mathrm{PIN2}}^{\mathrm{upper\ side}}}{P_{\mathrm{PIN2}}^{\mathrm{lower\ side}}}$ plays the major role in setting up the IAA asymmetry $\frac{\mathrm{IAA}^{\mathrm{lower}}}{\mathrm{IAA}^{\mathrm{upper}}}$, and absolute values of $P_{\mathrm{PIN2}}^{\mathrm{upper}}$ and $P_{\mathrm{PIN2}}^{\mathrm{lower}}$ do not markedly influence the resulting $\frac{\mathrm{IAA}^{\mathrm{lower}}}{\mathrm{IAA}^{\mathrm{upper}}}$ (compare result for $k = 0.5$ Supplementary Fig.16e with the main model Fig. 8b).

In addition, a definition of PIN2 asymmetry by Supplementary Eq. 15 & 16 allows to assess IAA distribution in a hypothetical case, in which PIN2 permeability on the upper side equals zero (Supplementary Fig.16g). It shows high IAA accumulation in meristem on the upper side, where there is no PIN2. However, it does not cause depletion of IAA from the epidermal cells of elongation zone on this side (Supplementary Fig.16g).

Supplementary Note 16 - Effect of overall PIN2 and AUX1 permeabilities and gravistimulation strength on auxin ratio $\frac{\mathrm{IAA}^{\mathrm{lower}}}{\mathrm{IAA}^{\mathrm{upper}}}$

In the main model we calculated $\frac{\mathrm{IAA}^{\mathrm{lower}}}{\mathrm{IAA}^{\mathrm{upper}}}(P_{\mathrm{PIN2}}^{\mathrm{initial}})$ for gravistimulation strengths ranging from $\frac{\mathrm{IAA\ flux}^{\mathrm{lower}}}{\mathrm{IAA\ flux}^{\mathrm{upper}}} = \frac{50\%}{50\%}$ (no gravistimulation) to $\frac{\mathrm{IAA\ flux}^{\mathrm{lower}}}{\mathrm{IAA\ flux}^{\mathrm{upper}}} = \frac{95\%}{5\%}$ (maximum gravistimulation achieved by placing all PIN3/7 to the lower side of the columella cells). Results of the simulation show that: 1) a maximum auxin ratio is achieved for any gravistimulation strength at an optimal PIN2 permeability $\bar{P}_{\mathrm{PIN2}}^{\mathrm{initial}} \cong 10$ (dimensionless PIN2 permeability normalized by $P_{\mathrm{PIN2}}^{0\ \mathrm{initial}} = 0.5\ \mathrm{\mu m/s}$: $\bar{P}_{\mathrm{PIN2}}^{\mathrm{initial}} = \frac{P_{\mathrm{PIN2}}^{\mathrm{initial}}}{P_{\mathrm{PIN2}}^{0\ \mathrm{initial}}}$) (Fig. 8h), and 2) a minimal gravistimulation strength (= 60% / 40%) is needed to achieve auxin ratios observed in eBL-treated roots ($\frac{\mathrm{IAA}^{\mathrm{lower}}}{\mathrm{IAA}^{\mathrm{upper}}} = 2.3$) for this optimal $\bar{P}_{\mathrm{PIN2}}^{\mathrm{initial}} = 10$. If PIN2 permeability differs from this optimal value, then even higher gravistimulation strengths are needed to reach $\frac{\mathrm{IAA}^{\mathrm{lower}}}{\mathrm{IAA}^{\mathrm{upper}}} = 2.3$ (Fig. 8h; red line). Auxin ratios observed under control conditions can be reached by lower gravistimulation strength (Fig. 8h, black line). Simulations show that a 2-to-100-fold increase in $\bar{P}_{\mathrm{PIN2}}^{\mathrm{initial}}$ is required for a change of $\frac{\mathrm{IAA}^{\mathrm{lower}}}{\mathrm{IAA}^{\mathrm{upper}}} = 1.62$ to $\frac{\mathrm{IAA}^{\mathrm{lower}}}{\mathrm{IAA}^{\mathrm{upper}}} = 2.3$, depending on gravistimulation strength and initial $\bar{P}_{\mathrm{PIN2}}^{\mathrm{initial}}$ (Fig. 8h).

Next, we investigated the dependence of the auxin ratio on the overall AUX1 permeability. $\frac{\mathrm{IAA}^{\mathrm{lower}}}{\mathrm{IAA}^{\mathrm{upper}}}(P_{\mathrm{AUX1}})$ is also non-monotonical, similar to $\frac{\mathrm{IAA}^{\mathrm{lower}}}{\mathrm{IAA}^{\mathrm{upper}}}(P_{\mathrm{PIN2}}^{\mathrm{initial}})$: 1) maximum auxin ratio is achieved at an optimal AUX1 permeability $\bar{P}_{\mathrm{AUX1}} \cong 2$, dimensionless AUX1 permeability normalized by $P_{\mathrm{AUX1}}^{0} = 0.35\,\frac{\mathrm{\mu m}}{\mathrm{s}}$: $\bar{P}_{\mathrm{AUX1}} = \frac{P_{\mathrm{AUX1}}}{P_{\mathrm{AUX1}}^{0}}$, for any gravistimulation strength (Supplementary Fig.17a), and 2) there is a minimal gravistimulation strength (= 60% / 40%)

needed to achieve the observed auxin ratio in eBL-treated roots ($\frac{\mathrm{IAA}^{\mathrm{lower}}}{\mathrm{IAA}^{\mathrm{upper}}} = 2.3$) for this optimal $P_{\mathrm{AUX1}} \cong 2$. If AUX1 permeability differs from this optimal value, then even higher gravistimulation strengths is needed to reach $\frac{\mathrm{IAA}^{\mathrm{lower}}}{\mathrm{IAA}^{\mathrm{upper}}} = 2.3$ (Supplementary Fig.17a, red line). Importantly, PIN2 asymmetry leads to diminished $\frac{\mathrm{IAA}^{\mathrm{lower}}}{\mathrm{IAA}^{\mathrm{upper}}}$ in case of gravistimulation compared to no asymmetry (compare graphs for $k = 1$ and $k = 0.5$) for any value of P_{AUX1}, thus AUX1 abundance does not influence this main conclusion of our model (Supplementary Fig.17b).

Next, Supplementary Fig.17c presents $\frac{\mathrm{IAA}^{\mathrm{lower}}}{\mathrm{IAA}^{\mathrm{upper}}}(P_{\mathrm{AUX1}})$ for the range of $P_{\mathrm{PIN2}}^{\mathrm{initial}}$ values. It shows, that PIN2/AUX1 permeability ratio governs the establishment of the auxin ratio. Thus, for example, 10 times higher PIN2 permeability ($P_{\mathrm{PIN2}}^{\mathrm{initial}} = 10$) moves an optimal AUX1 permeability, at which maximum $\frac{\mathrm{IAA}^{\mathrm{lower}}}{\mathrm{IAA}^{\mathrm{upper}}}$ is achieved, to higher values ($P_{\mathrm{AUX1}}^{\mathrm{optimal}} \cong 2 \rightarrow P_{\mathrm{AUX1}}^{\mathrm{optimal}} \cong 4$). On the contrary, a 10 times lower PIN2 permeability ($P_{\mathrm{PIN2}}^{\mathrm{initial}} = 0.1$) moves an optimal AUX1 permeability, at which maximum $\frac{\mathrm{IAA}^{\mathrm{lower}}}{\mathrm{IAA}^{\mathrm{upper}}}$ is achieved, to lower values ($P_{\mathrm{AUX1}}^{\mathrm{optimal}} \cong 2 \rightarrow P_{\mathrm{AUX1}}^{\mathrm{optimal}} \cong 0.4$). Thus, $\frac{\mathrm{IAA}^{\mathrm{lower}}}{\mathrm{IAA}^{\mathrm{upper}}}$ depends on the relative permeabilities of PIN2 and AUX1 in a nonlinear way, and can be predicted precisely only if values of P_{AUX1} and P_{PIN2} are known. Nevertheless, for a given P_{AUX1}, Supplementary Fig.17c predicts how $\frac{\mathrm{IAA}^{\mathrm{lower}}}{\mathrm{IAA}^{\mathrm{upper}}}$ changes with P_{PIN2} and *vice versa*.

Finally, Supplementary Fig. 17d presents an exemplified simulation with control root conditions as follows: $\bar{P}_{\mathrm{PIN2}}^{\mathrm{initial}} = 0.5$, $\bar{P}_{\mathrm{AUX1}} = 0.7$, $k = 0.81$ (bar #1) as a starting parameter set and shows how $\bar{P}_{\mathrm{PIN2}}^{\mathrm{initial}}$, $\bar{P}_{\mathrm{AUX1}}$ and PIN2 asymmetry (k) changes influence increase in $\frac{\mathrm{IAA}^{\mathrm{lower}}}{\mathrm{IAA}^{\mathrm{upper}}}$ in this particular example, which is within physiologically relevant parameter range. Important conclusion is that the effect of PIN2 asymmetry (k=0.81; bar #1 vs k=1; bar #6) is higher than effects of a twofold increase in $\bar{P}_{\mathrm{PIN2}}^{\mathrm{initial}}$ (bar #2) or $\bar{P}_{\mathrm{AUX1}}$ (bar #3) alone. $\frac{\mathrm{IAA}^{\mathrm{lower}}}{\mathrm{IAA}^{\mathrm{upper}}}$ values in eBL-treated roots can be achieved by elimination of PIN2 asymmetry, together with a mild increase in $\bar{P}_{\mathrm{PIN2}}^{\mathrm{initial}}$ and $\bar{P}_{\mathrm{AUX1}}$ (bar #10). Alternitevely, simultaneous threefold increase in $\bar{P}_{\mathrm{PIN2}}^{\mathrm{initial}}$ and $\bar{P}_{\mathrm{AUX1}}$ is required for that, if PIN2 asymmetry is unchanged, when compared to the control (bar #11), which is much higher increase, than measured experimentally in Supplementary Fig. 15c.

Supplementary Note 17 - Main conclusions

1) A decrease in PIN2 abundance on one side of the root results in an increase of $[IAA]_{cell}$ concentration in epidermal cells on the respective side. Consequently, the IAA ratio at the lower/upper sides is inversely proportional to the PIN2 ratio: $\frac{\mathrm{IAA}^{\mathrm{lower}}}{\mathrm{IAA}^{\mathrm{upper}}} \cong \frac{P_{\mathrm{PIN2}}^{\mathrm{upper\ side}}}{P_{\mathrm{PIN2}}^{\mathrm{lower\ side}}}$. This relation does not depend on the root shape, boundary conditions or transport properties of the neighboring cells and depends only on PIN2 abundance as far as AUX1 and other transporters have the same abundance in the cells for which ratio is calculated.

2) The coefficient of this proportionality depends on the initial IAA ratio; therefore, effects are higher in gravistimulated roots: $\frac{\mathrm{IAA}^{\mathrm{lower}}}{\mathrm{IAA}^{\mathrm{upper}}} \cong \frac{\mathrm{IAA}_{\mathrm{initial}}^{\mathrm{lower}}}{\mathrm{IAA}_{\mathrm{initial}}^{\mathrm{upper}}} \cdot \frac{P_{\mathrm{PIN2}}^{\mathrm{upper\ side}}}{P_{\mathrm{PIN2}}^{\mathrm{lower\ side}}}$ (Fig. 8g).

3) The effect of PIN2 asymmetry on $\frac{\mathrm{IAA}^{\mathrm{lower}}}{\mathrm{IAA}^{\mathrm{upper}}}$ is opposite to the effect of gravistimulus-mediated PIN3/PIN7 relocation (which increases the ratio $\frac{\mathrm{IAA}^{\mathrm{lower}}}{\mathrm{IAA}^{\mathrm{upper}}}$).

4) The effect of PIN2 asymmetry on $\frac{\mathrm{IAA}^{\mathrm{lower}}}{\mathrm{IAA}^{\mathrm{upper}}}$ is lower than that of PIN3/PIN7 asymmetry in a physiologically relevant range. Thus, PIN2 asymmetry does not result in an equilibration of IAA concentrations on the upper and lower sides of gravistimulated roots. Instead, maximum observed PIN2 asymmetries ($k = 0.81$; Fig. 6e) reduce the ratio $\frac{\mathrm{IAA}^{\mathrm{lower}}}{\mathrm{IAA}^{\mathrm{upper}}}$ in the gravistimulated root by ~ 20% (Fig. 8g; orange line).

5) The maximum effect of PIN2 asymmetry on $\frac{\mathrm{IAA}^{\mathrm{lower}}}{\mathrm{IAA}^{\mathrm{upper}}}$ is achieved, if PIN2 permeability is in the order of 0.1 - 1 um/s (Supplementary Fig.16a) and AUX1 permeability is in the order of 0.1 - 3 um/s (Supplementary Fig.17b). We conclude that the control roots might display this maximum theoretical effect of PIN2 asymmetry on $\frac{\mathrm{IAA}^{\mathrm{lower}}}{\mathrm{IAA}^{\mathrm{upper}}}$ *in vivo*, because permeability values, reported in literature (see Supplementary Table 1) and used in the model as default values, $P_{\mathrm{PIN2}}^{0\ \mathrm{initial}}$and P_{AUX1}^{0} , are within these ranges.

6) The time frame required for $[IAA]_{cell}$ establishing steady-state is ~1000 sec (Fig. 8ef), which is much shorter than the full process of gravitropic bending. This argues against long-term effects of PIN2 asymmetry on $[IAA]_{cell}$. Once PIN2 symmetry is reestablished, $\frac{\mathrm{IAA}^{\mathrm{lower}}}{\mathrm{IAA}^{\mathrm{upper}}}$ ratio should also return to $\frac{\mathrm{IAA}^{\mathrm{lower}}}{\mathrm{IAA}^{\mathrm{upper}}} = 1$ within a short period of time.

SUPPLEMENTARY TABLES

Supplementary Table 1. Parameters values*, ranges and expressions used in the model. *Parameter values used as default are denoted by "0" superscript.

Parameter	Notation	Value	Reference value/ Expression	Use in the model setups
Permeability of influx carriers	P^0_{AUX1}	0.35 µm/s	$P_{AUX1} \cdot A_2 = 1.5 \pm 0.3\ \mu\mathrm{m/s}$ => $P_{AUX1} = 0.35 \pm 0.07\ \mu m/s$ [7]	All models
Permeability of PIN1 efflux carriers	P^0_{PIN1}	0.5 µm/s	$P_{\mathrm{PIN1}} \cdot B_3 = 2\mu\mathrm{m/s}$ => $P_{PIN1} = 0.43\ \mu\mathrm{m/s}$ [7]	All models
Permeability of PIN3/PIN7 efflux carriers in columella cells	$P^0_{PIN3/PIN7}$	0.5 µm/s	$= P^0_{PIN1}$	All models, No gravistimulation
Initial permeability of PIN2 before onset of asymmetry	$P^{0\ \mathrm{initial}}_{\mathrm{PIN2}}$	0.5 µm/s	$P_{PIN2} \cdot B_3 = 2\frac{\mu m}{s} => P_{PIN2} = 0.43\ \mu m/s$ [7]	All models
Sweep of initial PIN2 permeability	$P^{\mathrm{initial}}_{\mathrm{PIN2}}$	range between 0.005 µm/s and 50 µm/s	range between $= P^{0\ \mathrm{initial}}_{\mathrm{PIN2}} \cdot 0.01$ $= P^{0\ \mathrm{initial}}_{\mathrm{PIN2}} \cdot 100$	Effect of $P^{\mathrm{initial}}_{\mathrm{PIN2}}$
Coefficient of PIN2 asymmetry	k	0.05 – 1.4	Fig. 7a, this study	All models
Reduced PIN2 permeability on the upper side	$P^{\mathrm{upper\ side}}_{\mathrm{PIN2}}$	$= P^{\mathrm{initial}}_{\mathrm{PIN2}} \cdot \sqrt{k}$		Main model, in case of asymmetry
Increased PIN2 permeability on the lower side	$P^{\mathrm{lower\ side}}_{\mathrm{PIN2}}$	$=\frac{P^{\mathrm{initial}}_{\mathrm{PIN2}}}{\sqrt{k}}$		Main model, in case of asymmetry
/ PIN2 permeability on the upper side	$P^{\mathrm{upper\ side}}_{\mathrm{PIN2}}$	$= P^{\mathrm{initial}}_{\mathrm{PIN2}} \cdot k$		Effect of *PIN2 depletion from the upper side only*
/ PIN2 permeability on the lower side	$P^{\mathrm{lower\ side}}_{\mathrm{PIN2}}$	$= P^{\mathrm{initial}}_{\mathrm{PIN2}}$		Effect of *PIN2 depletion from the upper side only*
Diffusive permeability for IAAH	P_{IAAH}	0.4 µm/s	$P_{IAAH} = 0.3 \pm 0.22\ \mu m/s$ [7]	All models
Permeability of non-PIN-efflux carriers	P_{NPE}	0 µm/s	$P_{NPE} \leq 0.3 \cdot P_{PIN}$ [3]	All models
pH in cytoplasm	pH_{cell}	5.3	[3]	All models
pH in the cell wall	pH_{wall}	7.2	[3]	All models
IAA dissociation constant	pK	4.8	[3]	All models
IAA diffusion in the cell	D_c	$600\ \mu\mathrm{m}^2/s$	[2]	All models
IAA diffusion in the media	D_m	$60\ \mu\mathrm{m}^2/s$	[2]	All models
IAA diffusion in the wall	D_w	$32\ \mu\mathrm{m}^2/s$	[6]	All models
Plasma membrane potential	V_m	-120 mV	[3]	All models
Cell wall thickness		0.2 µm	rounded from [3]	All models

SUPPLEMENTARY FIGURES

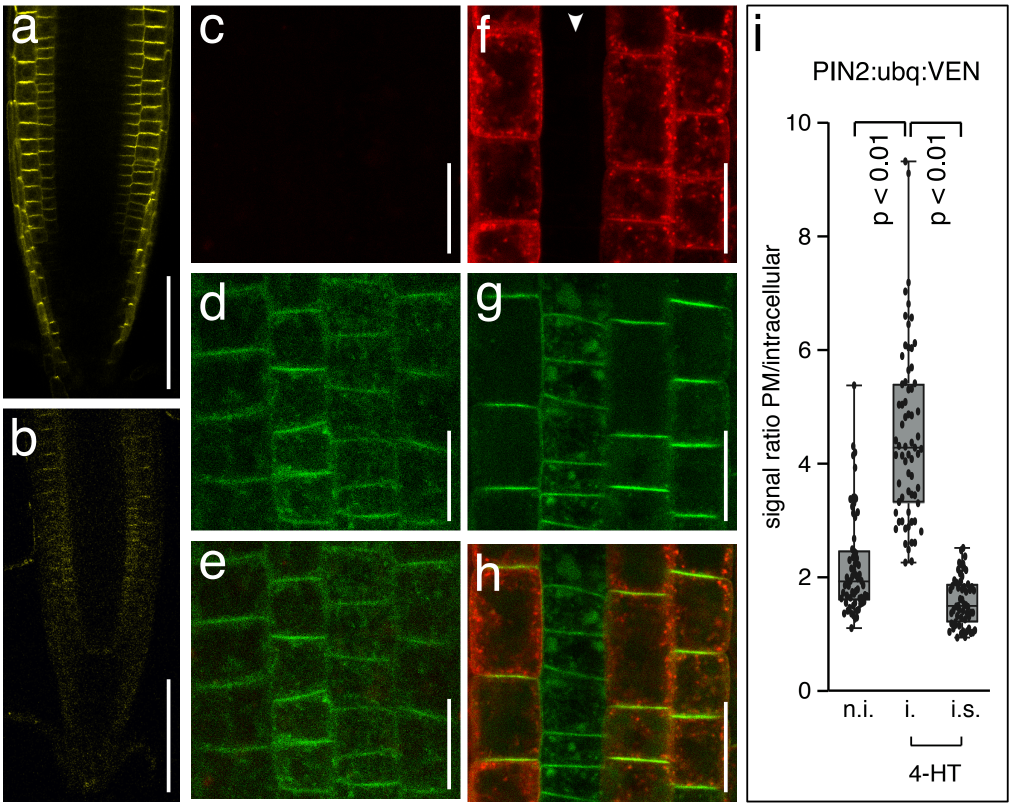

Supplementary Figure 1. Clathrin-mediated endocytosis of PIN2:ubq:VEN. **a,b)** Optical section through root meristems of *eir1-4 PIN2p::PIN2:VEN* (a) and *eir1-4 PIN2p::PIN2:ubq:VEN* (b) seedlings at 6 DAG. **c-h)** Co-expression analysis of *pINTAM>>RFP:HUB* (c,f) and *PIN2p::PIN2:ubq:VEN* (d,g) with merged images (e,h) in epidermal cells of non-induced (c,d,e) and seedlings on 2 µM 4-hydroxytamoxifen for 24 hours (f,g,h). White arrowhead labels a cell file in which *RFP:HUB* remains silenced under induced conditions. **i)** PIN2:ubq:VEN signal ratios between PM and vacuoles in *pINTAM>>RFP:HUB* non-induced (n.i.), induced (i.), and induced but silenced (i.s.) seedlings; 4-HT (4-hydroxytamoxifen). 57-67 root epidermal cells in 6 roots were tested for each dataset and analyzed by one-way ANOVA with post-hoc Tukey HSD test (p-values as indicated, no significant difference was detected between ‘n.i.’ and ‘i.s.’ samples). Box plot represents the entire range of outliers (whiskers) and first and third quartiles (grey boxes); center line: median. Scale bars: a,b = 50 µm; c-h = 10 µm. Source data are provided as Source Data file.

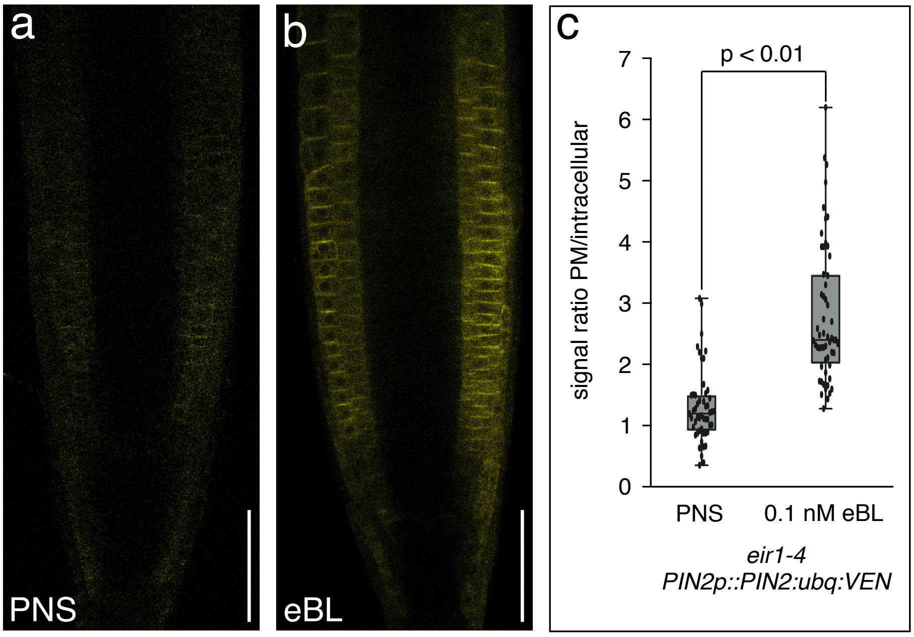

Supplementary Figure 2. *PIN2p::PIN2:ubq:VEN* expression upon germination on 0.1 nM eBL. **a,b)** *eir1-4 PIN2p::PIN2:ubq:VEN* root meristem at 5 DAG, germinated on PNS (a) and on PNS supplemented with 0.1 nM eBL (b). **c)** PIN2:ubq:VEN signal ratios between PM and intracellular space in *eir1-4 PIN2p::PIN2:ubq:VEN*. In total, 50/53 root meristem epidermis cells in 10 roots were tested for each dataset. Two-tailed *t*-test analysis of resulting values revealed a significant difference. Whiskers in the box plot represent the entire range of outliers obtained in the datasets; grey boxes display first and third quartiles, center line: median. Size bars: a,b = 50 µm. Source data are provided as Source Data file.

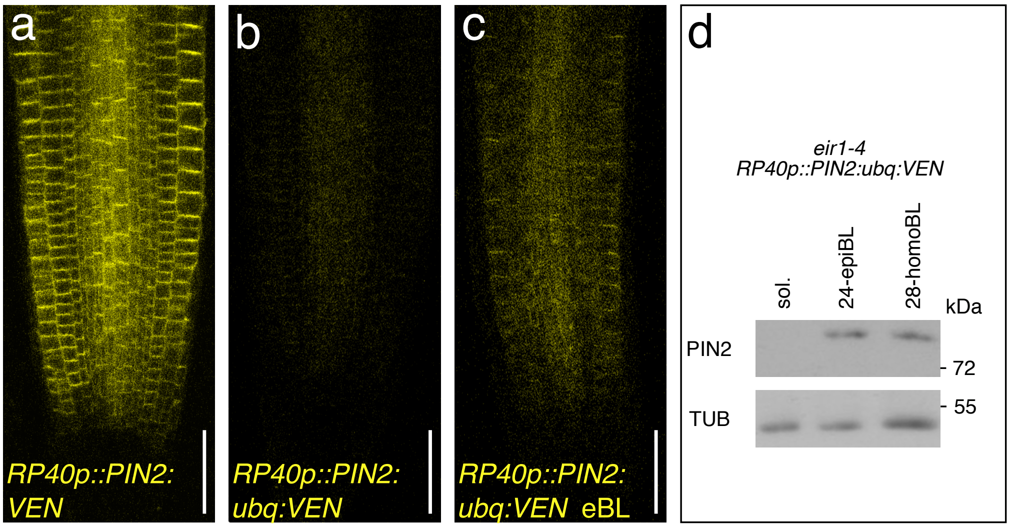

Supplementary Figure 3. Comparison of *PIN2:VEN* and *PIN2:ubq:VEN* expressed under control of the *RP40* promoter. **a-c)** Venus signals in *eir1-4 RP40p::PIN2:VEN* (a) and *eir1-4 RP40p:: PIN2:ubq:VEN* (b,c) root tips, grown on PNS (a,b), or incubated for 16 hours in presence of 100 nM eBL (c). **d)** Western blots performed with *eir1-4 RP40p:: PIN2:ubq:VEN* membrane protein extracts, probed with anti-PIN2. Samples were incubated for 16 hours in presence of 100 nM 24-epiBL and 100 nM 28-homoBL, respectively. Mock-treated material (sol.) served as control. Size bars: a-c = 30 µm. Anti-α-tubulin (TUB) was used as loading control. Source data are provided as Source Data file.

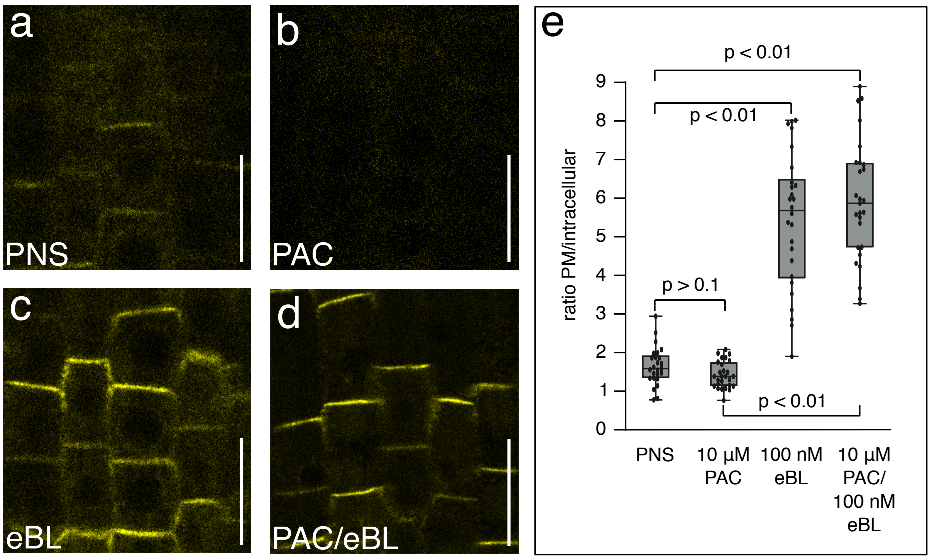

Supplementary Figure 4. Analysis of GA-brassinolide crosstalk in the regulation of *PIN2p::PIN2:ubq:VEN*. **a-d)** Venus signals in *eir1-4 PIN2p::PIN2:ubq:VEN* root epidermis cells at 5 DAG, grown on PNS (a), incubated o/n in presence of 10 µM paclobutrazole (PAC, b), 100 nM eBL (c), and 10 µM paclobutrazole/100 nM eBL (PAC/eBL, d). **e)** PIN2:ubq:VEN signal ratios between PM and intracellular space in *eir1-4 PIN2p::PIN2:ubq:VEN*. In total, 25-27 root meristem epidermis cells in 5 roots were tested for each dataset. p-values obtained by One-way ANOVA with post-hoc Tukey HSD test are indicated. Whiskers represent the entire range of outliers obtained in the datasets; grey boxes display first and third quartiles, center line: median. Size bars: a,b = 10 µm. Source data are provided as Source Data file.

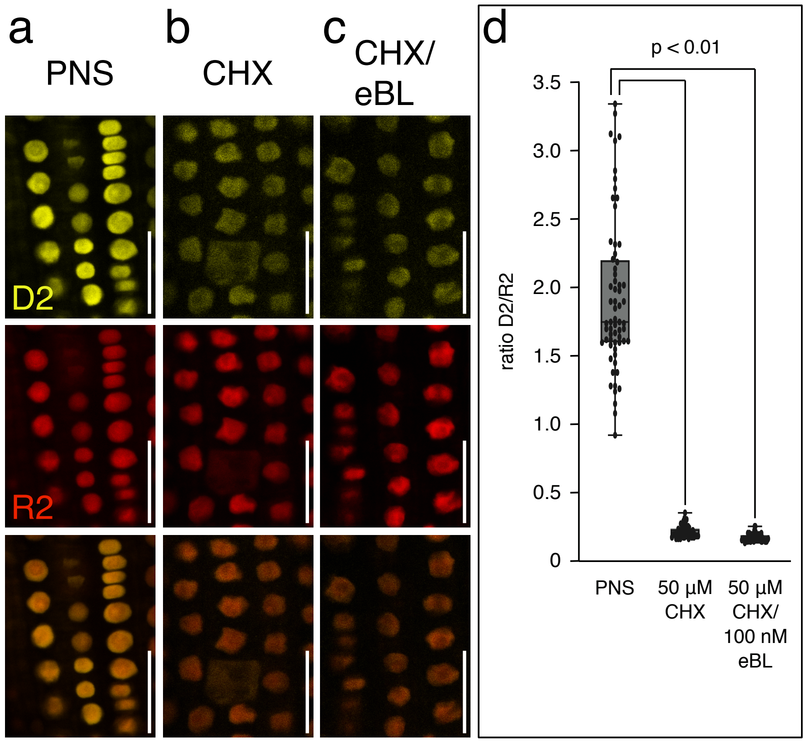

Supplementary Figure 5. *R2D2* expression in 5-day-old *Arabidopsis* root meristem cells upon translational inhibition. **a-c)** D2 (yellow) and R2 (red) signals in seedlings incubated on PNS (a), PNS supplemented with 50 µM CHX for 5.5 hours (b), PNS supplemented with 50 µM CHX for 30 minutes followed by coincubation in presence of 50 µM CHX and 100nM eBL for another 5 hours (c). Bottom panels display merged channels. **d)** Box plot, displaying the ratio of D2 and R2 signals upon translational inhibition. In total, 57-63 epidermis root meristem cells in 8 roots were tested for each dataset. One-way ANOVA with post-hoc Tukey HSD test was used to determine p-values. Whiskers represent the entire range of outliers obtained in the datasets; grey boxes display first and third quartiles, center line: median. Size bars: a-c = 20 µm. Source data are provided as Source Data file.

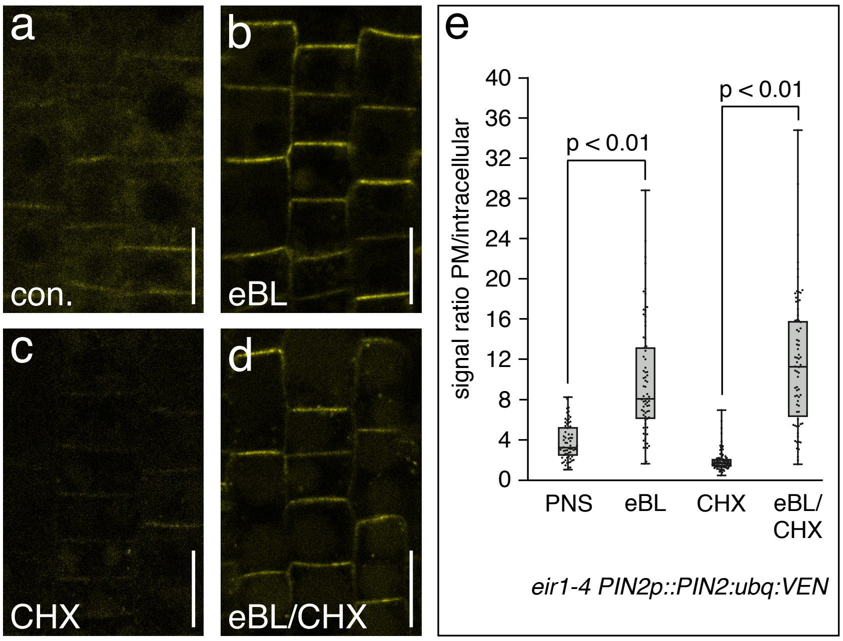

Supplementary Figure 6. Translational inhibition and expression of PIN2:ubq:VEN. **a-d)** Venus signals in 5-day-old *eir1-4 PIN2p::PIN2:ubq:VEN* root meristem epidermis cells, grown on PNS (a), supplemented with 100 nM eBL for 18 hours (b), incubated in presence of 50 µM CHX for 150 minutes (c), and supplemented with 100 nM eBL for 16 hours followed by co-incubation in presence of 50 µM CHX and 100 nM eBL for another 150 minutes (d). **e)** PIN2:ubq:VEN signal ratios between PM and intracellular space in *eir1-4 PIN2p::PIN2:ubq:VEN*. In total, 67-96 root meristem epidermis cells of 6-10 roots were tested for each dataset. Two-tailed *t*-test analysis of resulting values revealed a significant difference in the distribution of Venus signals, when comparing PNS-grown and eBL-treated seedlings as well as when comparing CHX-treated and eBL/CHX-treated material. Whiskers in the box plot represent the entire range of outliers obtained in the datasets; grey boxes display first and third quartiles; center line: median. Size bars: a-d = 10 µm. Source data are provided as Source Data file.

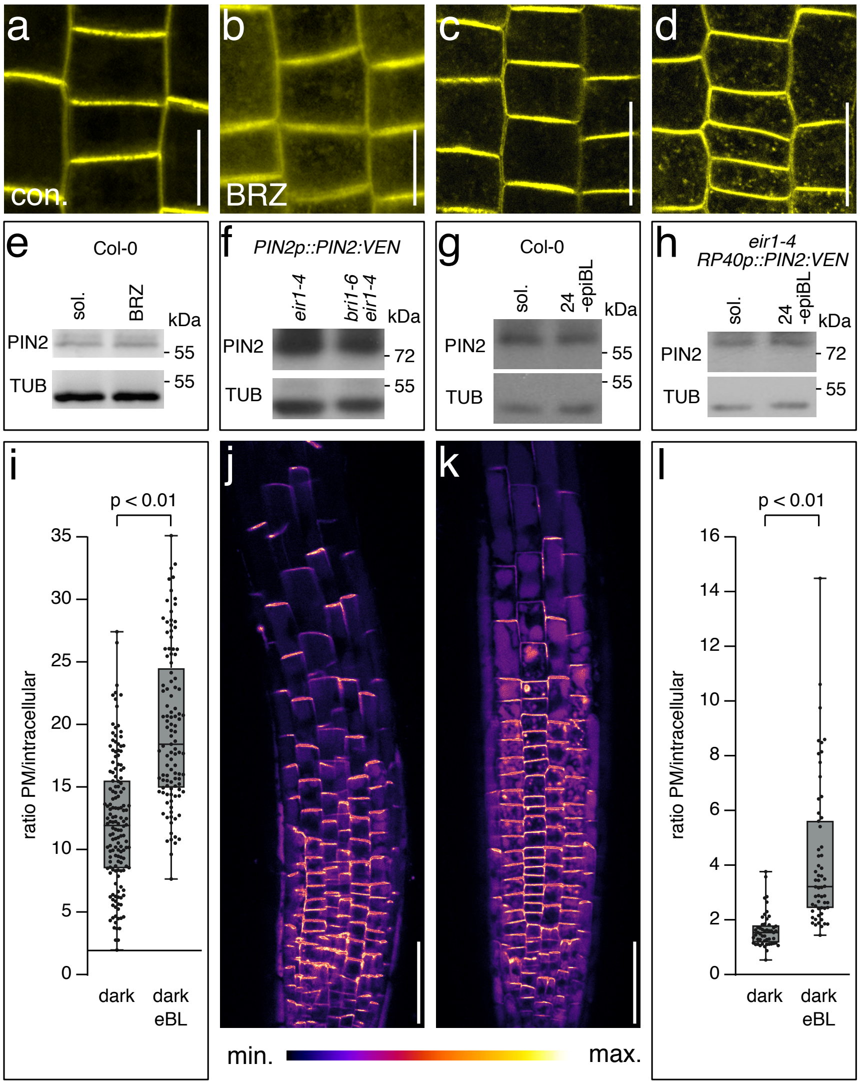

Supplementary Figure 7. Wild type *PIN2* and *PIN2p::PIN2:VEN* expression in response to altered brassinolide homeostasis and signaling **a,b)** PIN2:Venus signals (yellow) in 6-day-old *eir1-4 PIN2p::PIN2:VEN* root meristem epidermis cells grown on PNS (a) or treated with 10 µM BRZ for 16 hours (b). **c,d)** PIN2:Venus distribution (yellow) in 6-day-old *eir1-4 PIN2p::PIN2:VEN* (c) and *bri1-6 eir1-4 PIN2p::PIN2:VEN* (d) root meristem epidermis cells. **e)** Western blot performed with Col0 membrane protein extracts, probed with anti-PIN2. 6-day-old seedlings were incubated in presence of 10 µM BRZ for 16 hours, and compared to mock-treated material (sol.). **f)** Western blot performed with *eir1-4 PIN2p::PIN2:VEN* and *bri1-6 eir1-4 PIN2p::PIN2:VEN* membrane protein extracts, probed with anti-PIN2. **g)** Western blot performed with Col0 wild type membrane protein extracts, probed with anti-PIN2. 6-day-old seedlings were incubated in presence of 100 nM eBL for 16 hours, and compared to mock-treated material (sol.). **h)** Western blot performed with *eir1-4 RP40p::PIN2:VEN* membrane

protein extracts, probed with anti-PIN2. 6-day-old seedlings were incubated in presence of 100 nM eBL for 16 hours, and compared to mock-treated material (sol.). **i)** PIN2m:Cherry signal ratios in root meristem epidermis cells of 6-day-old dark-incubated *eir1-4 PIN2p::PIN2:mCherry* grown on control medium, or on medium supplemented with 100 nM eBL for 3 hours. Signal ratios between PM and intracellular space were determined in 109/150 trichoblast cells in 8 roots. Two-tailed *t*-test analysis of resulting values revealed a significant difference. **j,k)** *eir1-4 PIN2p::PIN2:mCherry* signal distribution in 6DAG primary root meristem after 16 hours dark incubation on 100 nM eBL (j) and control medium (k). **l)** PIN2m:Cherry signal ratios in root meristem epidermis cells of 6DAG *eir1-4 PIN2p::PIN2:mCherry* grown on control medium, or on medium supplemented with 100 nM eBL and dark-incubated for 16 hours. Signal ratios between PM and intracellular space were determined in 58/51 trichoblast cells in 6 roots for each dataset. Two-tailed *t*-test analysis of resulting values revealed a significant difference. Whiskers in the box plots represent the entire range of outliers obtained in the datasets; grey boxes display first and third quartiles, center line: median. Size bars: a,b = 10 µm; c,d = 20 µm; j,k = 50 µm. Anti-α-tubulin (TUB) was used as loading control on all Western blots. Source data are provided as Source Data file.

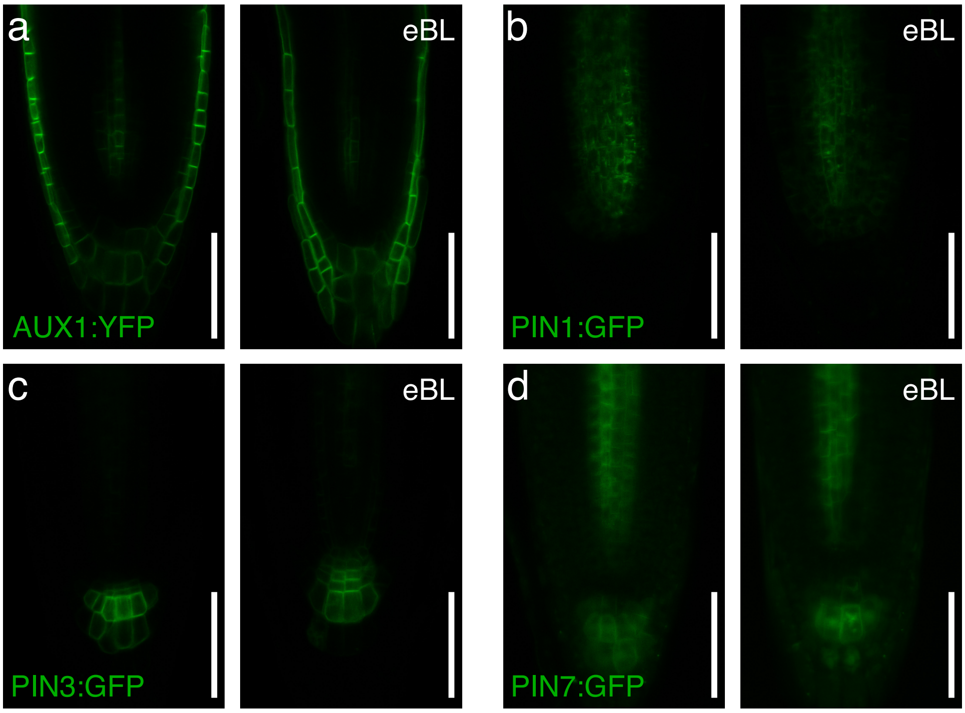

Supplementary Figure 8. Expression of AUX1 and PIN reporter proteins in brassinolide-treated seedlings (green). **a-d)** 6-day-old seedlings expressing the indicated reporter constructs, were transferred on medium containing 100 nM eBL, or on control medium lacking brassinolide, and incubated for another 16 hours. Roots expressing *AUX1p::AUX1:YFP* (a), *PIN1p::PIN1:GFP* (b), *PIN3p::PIN3:GFP* (c) and *PIN7p::PIN7:GFP* (d) were viewed at the CLSM. Control roots are displayed on the left, whereas eBL-treated individuals are shown on the right. Size bars: a-d = 50 μm.

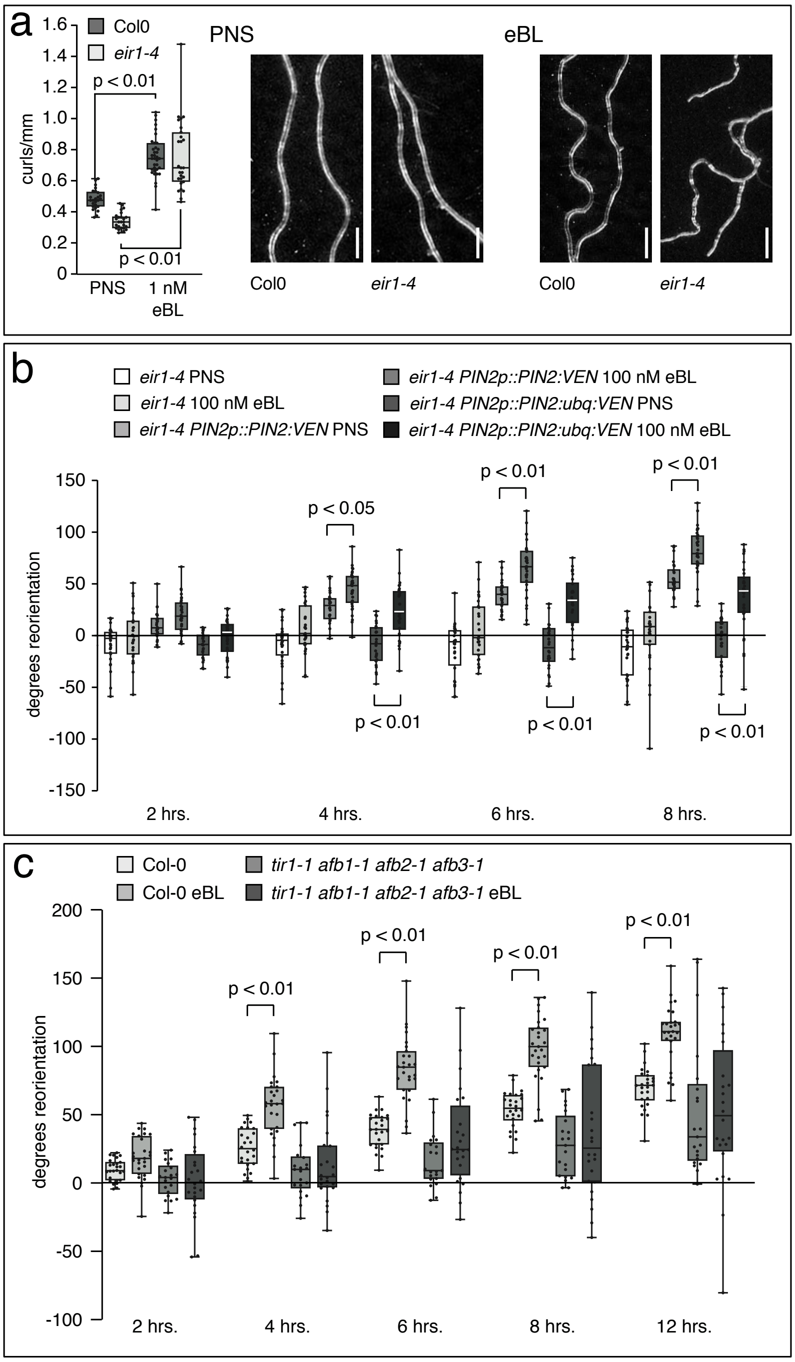

Supplementary Figure 9. Crosstalk between brassinolide and auxin in directional root growth. **a)** Root curling in 6 DAG Col0 and *eir1-4* seedlings germinated on PNS or on 1 nM eBL. 25-35 roots were analyzed for each dataset. Two-tailed *t*-test analysis resulted in p-values as indicated. Inlay: Root phenotypes under conditions tested. **b)** Kinetics of root reorientation of 6 DAG *eir1-4, eir1-4 PIN2p::PIN2:VEN and eir1-4 PIN2::PIN2:ubq:VEN* primary roots incubated on the indicated growth medium. 26-33 roots were analyzed for each dataset. One-way ANOVA with post-hoc Tukey HSD test was used to determine p-values. **c)** Kinetics of root reorientation of 6 DAG Col0 and *tir1-1 afb1-1 afb2-1 afb3-1* primary roots incubated on the indicated growth medium. 21-27 roots were analyzed for each dataset. One-

way ANOVA with post-hoc Tukey HSD test was used to determine p-values. Whiskers in the box plots represent the entire range of outliers obtained in the datasets; grey boxes display first and third quartiles; center line: median. Size bars: a = 1 mm. Source data are provided as Source Data file.

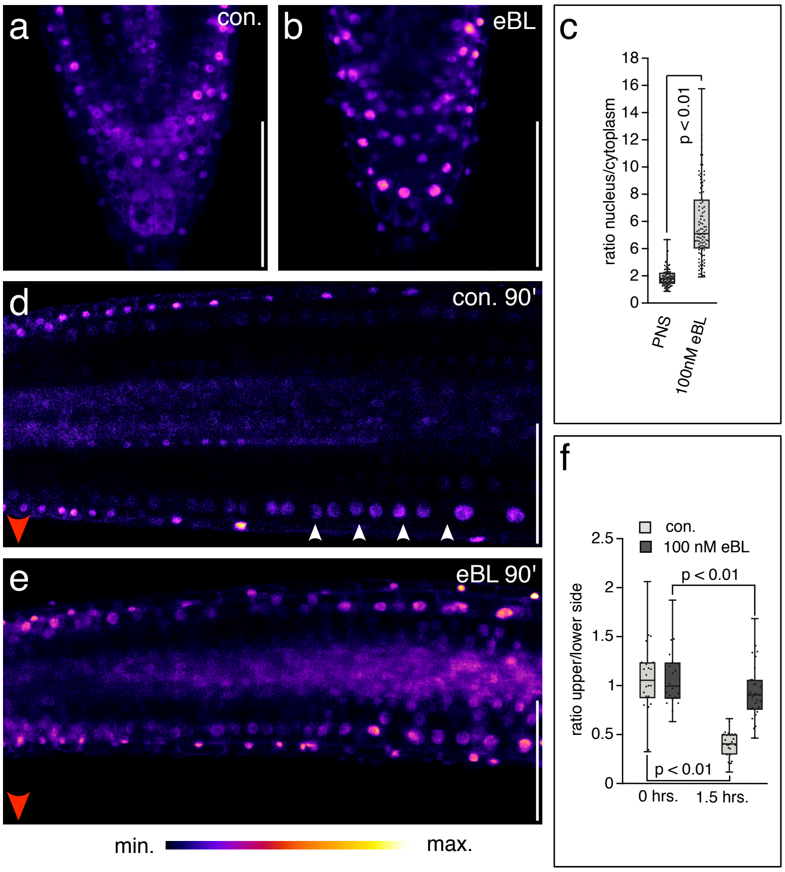

Supplementary Figure 10. Expression of brassinolide responsive *35S::BES1:GFP*. **a,b)** Distribution of BES1:GFP signals in columella root cap cells on control medium (a) and medium supplemented with 100 nM eBL for 16 hours. **c)** Nucleus/cytoplasm signal ratio in *35S::BES1:GFP* columella root cap cells at 6 DAG. 100/102 cells were analyzed in 7 roots for each dataset. Two-tailed *t*-test analysis resulted in p-values as indicated. **d,e)** BES1:GFP signal ratios at the upper vs. lower side of 6-days-old horizontally positioned roots on control medium (d) and on medium supplemented with 100 nM eBL for 16 hours (e), after 90 minutes of gravistimulation. Red arrowheads indicate direction of the gravity vector, white arrowheads point to formation of a BES1:GFP signal gradient in root epidermis cells. **f)** BES1:GFP signal ratios at the upper vs. lower side of gravistimulated root meristems, grown on control medium (con.) or pre-treated with 100 nM eBL for 16 hours. 20-30 roots were analyzed for each dataset. Two-tailed *t*-test analysis resulted in p-values as indicated. Size bars: a,b = 40 µm, d,e = 50 µm. Source data are provided as Source Data file.

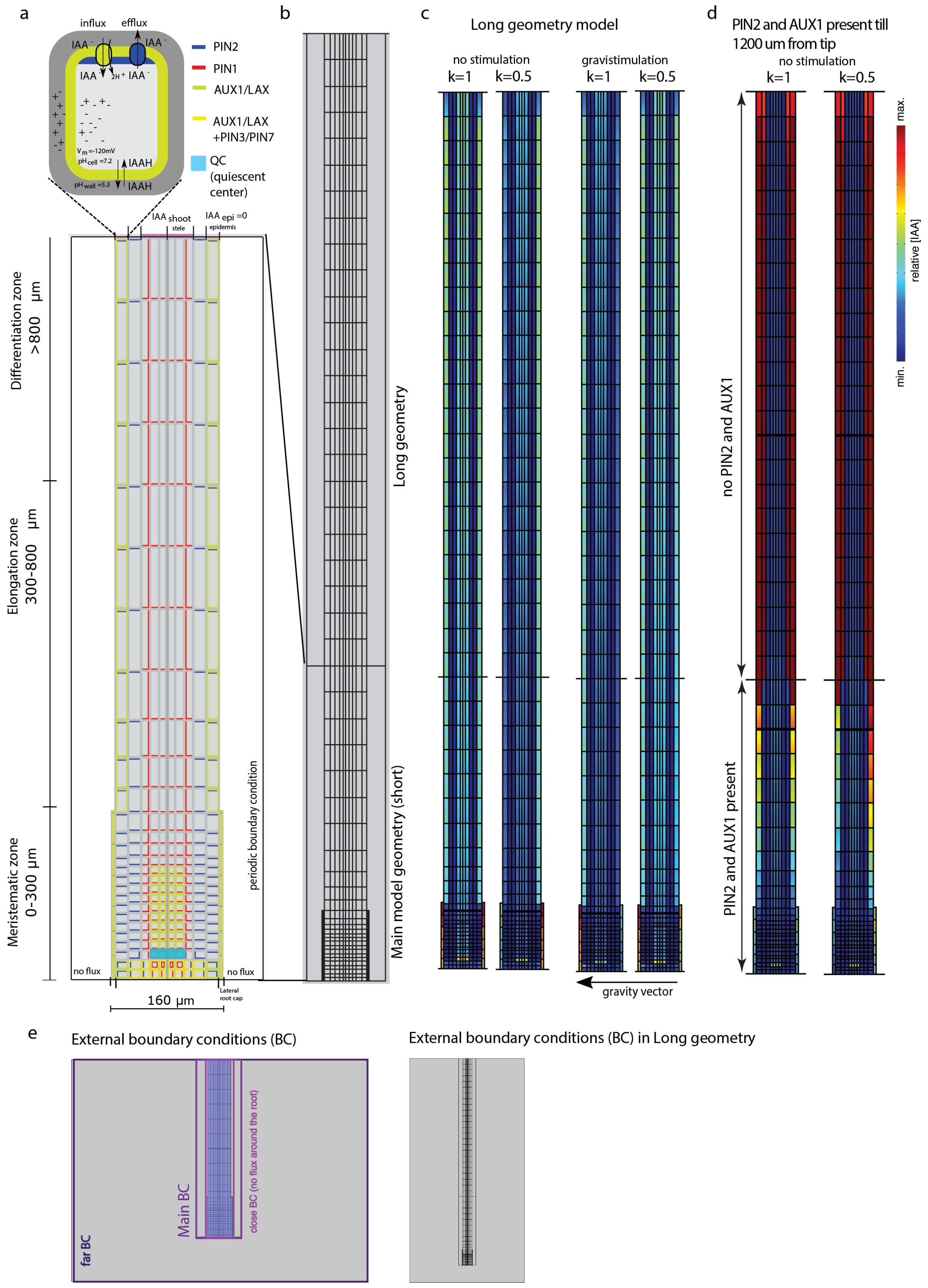

Supplementary Figure 11. Model description and simulated auxin distributions: effect of root geometry. **a)** Main model geometry with color-coded localization of influx (AUX1, LAX) and

efflux (PIN1, PIN2, PIN3/PIN7) auxin transporters. The auxin source is at the outer membrane of the uppermost cells in the stele, where IAA_{shoot} = 1; the auxin sink is above the epidermis cell files, where IAA_{epi} = 0. Inset: scheme of auxin transport between the wall and cytoplasm of one cell. **b)** Elongated model geometry (3500 μm long, see Supplementary Note 2.2). **c)** IAA distribution in the elongated geometry. Roots are presented in a vertical orientation, with the "lower side" on the left, and the "upper side" on the right (see gravity vector). Case of no stimulation (left): $\frac{\mathrm{IAA}^{\mathrm{lower}}}{\mathrm{IAA}^{\mathrm{upper}}}$ = 1 at no asymmetry $\frac{P_{\mathrm{PIN2}}^{\mathrm{upper\ side}}}{P_{\mathrm{PIN2}}^{\mathrm{lower\ side}}} = k = 1$; $\frac{\mathrm{IAA}^{\mathrm{lower}}}{\mathrm{IAA}^{\mathrm{upper}}}$ < 1 at $k = 0.5$. Case of gravistimulation (right; gravistimulation strength $\frac{70\%}{30\%}$) $\frac{\mathrm{IAA}^{\mathrm{lower}}}{\mathrm{IAA}^{\mathrm{upper}}}$ > 1 at no asymmetry $k = 1$; $\frac{\mathrm{IAA}^{\mathrm{lower}}}{\mathrm{IAA}^{\mathrm{upper}}}$~=1 at $k = 0.5$. **d)** Simulated IAA distribution with PIN2 and AUX1 absent from the upper differentiation zone. No stimulation: $\frac{\mathrm{IAA}^{\mathrm{lower}}}{\mathrm{IAA}^{\mathrm{upper}}}$ = 1 at no asymmetry $k = 1$; $\frac{\mathrm{IAA}^{\mathrm{lower}}}{\mathrm{IAA}^{\mathrm{upper}}}$ < 1 at $k = 0.5$. **e)** Position of external boundaries in the model variants.

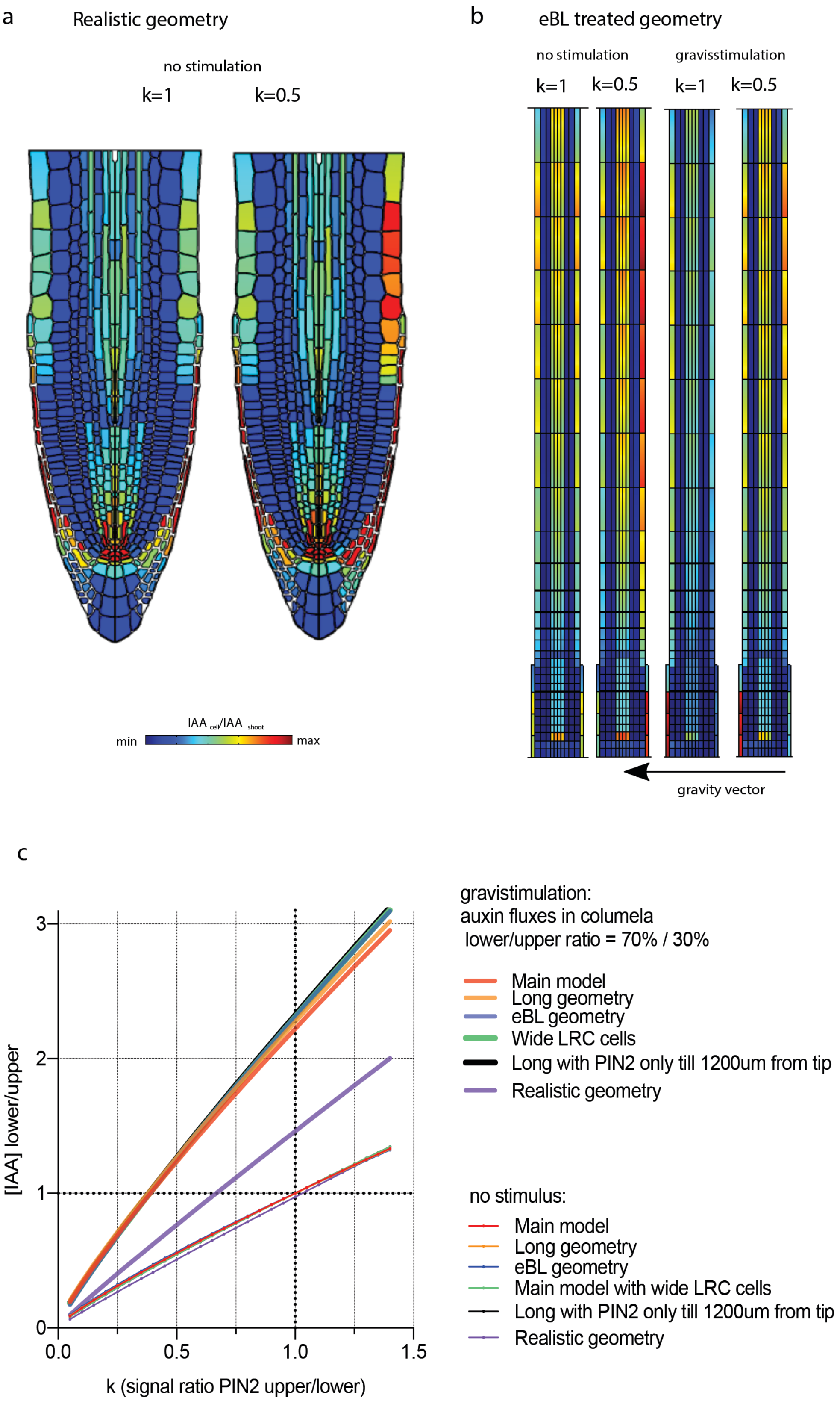

Supplementary Figure 12. Effect of root geometry on auxin distributions (continuation). **a)** IAA distribution when using a realistic root geometry. Case of no stimulation: $\frac{\text{IAA}^{\text{lower}}}{\text{IAA}^{\text{upper}}}$=1 at no

asymmetry $\frac{P_{\text{PIN2cortical}}^{\text{upper}}}{P_{\text{PIN2cortical}}^{\text{lower}}} = k = 1$; $\frac{\text{IAA}^{\text{lower}}}{\text{IAA}^{\text{upper}}}$ < 1 at $k = 0.5$. **b)** IAA distribution in the eBL-treated root geometry. Case of no stimulation(left): $\frac{\text{IAA}^{\text{lower}}}{\text{IAA}^{\text{upper}}}$ = 1 at no asymmetry $k = 1$; $\frac{\text{IAA}^{\text{lower}}}{\text{IAA}^{\text{upper}}}$ < 1 at $k = 0.5$. Case of gravistimulation (right; gravistimulation strength: $\frac{\text{IAA flux}^{\text{lower}}}{\text{IAA flux}^{\text{upper}}} = \frac{70\%}{30\%}$). $\frac{\text{IAA}^{\text{lower}}}{\text{IAA}^{\text{upper}}}$ > 1 at no asymmetry $k = 1$; $\frac{\text{IAA}^{\text{lower}}}{\text{IAA}^{\text{upper}}}$ < 1 at $k = 0.5$. Roots are presented in a vertical orientation, with the "lower side" on the left, and the "upper side" on the right (see gravity vector). **c)** $\frac{\text{IAA}^{\text{lower}}}{\text{IAA}^{\text{upper}}}(k)$ for different model variants for "no stimulation" and "gravistimulation" cases. $\frac{\text{IAA}^{\text{lower}}}{\text{IAA}^{\text{upper}}}$ is proportional to $\frac{P_{\text{PIN2cortical}}^{\text{upper}}}{P_{\text{PIN2cortical}}^{\text{lower}}} = k$ for all models.

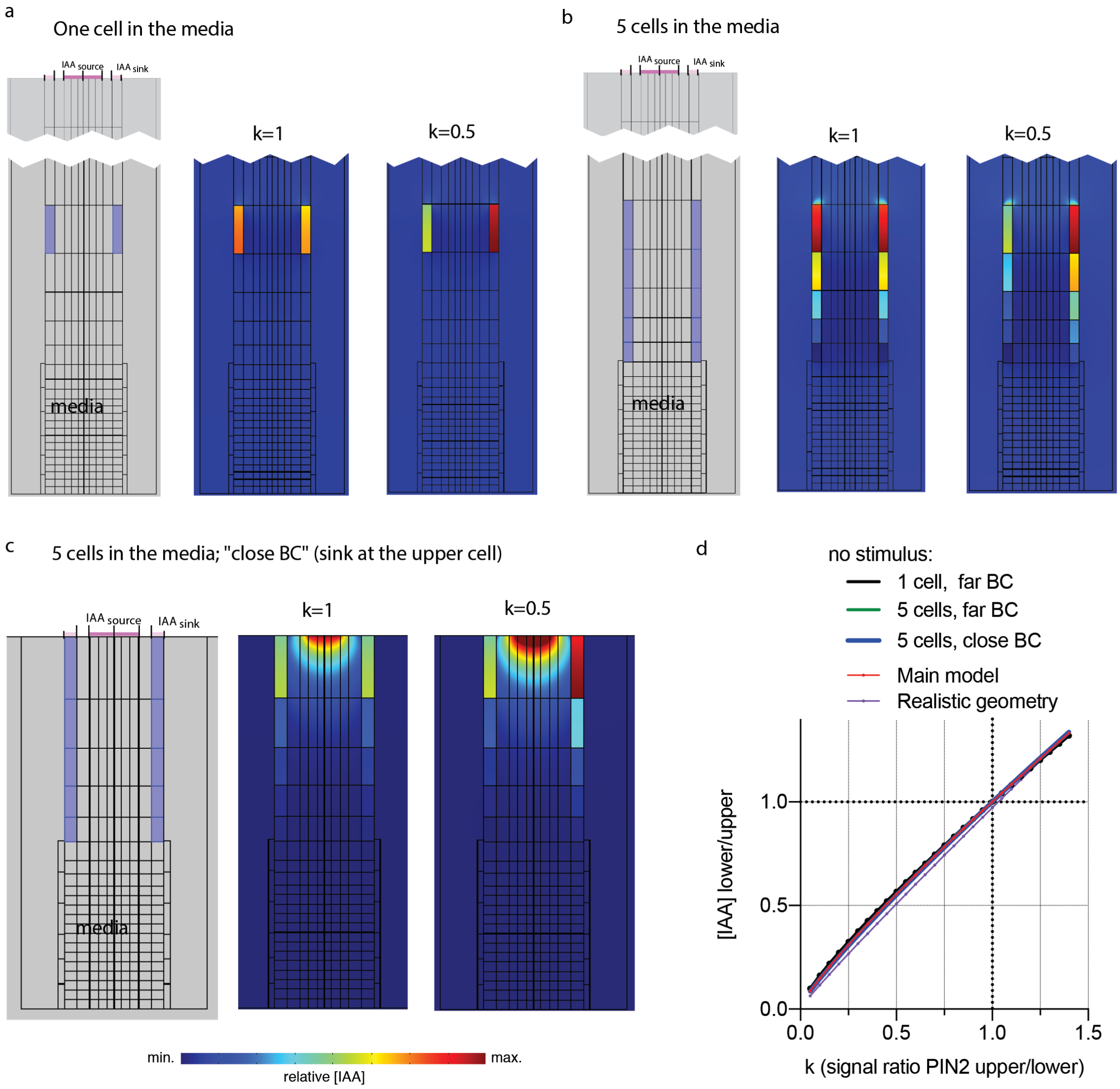

Supplementary Figure 13. Results of simplified models of 1 or 5 cells. **a)** Model of one cell in media. Left: scheme of elongated geometry, only lower and upper part shown; intracellular domains (blue); extracellular space/media (grey); source/sink BC (pink). Middle: steady state relative $[IAA]_{cell}$ at $\frac{P_{\text{PIN2}}^{\text{right}}}{P_{\text{PIN2}}^{\text{left}}} = 1$, permeabilities as in the main model P_{AUX1}^{0}, $P_{\text{PIN2}}^{0\ \text{initial}}$. Right: steady-state relative $[IAA]_{cell}$ at PIN2 asymmetry with higher PIN2 in the left cell ($\frac{P_{\text{PIN2}}^{\text{right}}}{P_{\text{PIN2}}^{\text{left}}} = \frac{P_{\text{PIN2}}^{0\ \text{initial}} \cdot \sqrt{k}}{P_{\text{PIN2}}^{0\ \text{initial}} / \sqrt{k}} = 0.5$); AUX1 permeability unchanged P_{AUX1}^{0}. **b)** Model of isolated column of 5 cells in media with "far BC". Left: scheme of elongated geometry, only lower and upper part shown; intracellular domains (blue); extracellular space/media (grey); source/sink BC (pink). Middle: steady-state relative $[IAA]_{cell}$ at $\frac{P_{\text{PIN2}}^{\text{right}}}{P_{\text{PIN2}}^{\text{left}}} = 1$, permeabilities as in the main model $P_{\text{AUX1}}^{0}, P_{\text{PIN2}}^{0\ \text{initial}}$. Right: steady-state relative $[IAA]_{cell}$ at PIN2 asymmetry with higher PIN2 in the left column

($\frac{P_{\mathrm{PIN2}}^{\mathrm{right}}}{P_{\mathrm{PIN2}}^{\mathrm{left}}} = 0.5$); AUX1 permeability unchanged P_{AUX1}^{0}. **c)** Model of isolated column of 5 cells in media with "close BC". Left: scheme of geometry with upper boundary close to the top cell; intracellular domains (blue); extracellular space/media (grey); source/sink BC (pink). Middle: steady-state relative $[\mathrm{IAA}]_{\mathrm{cell}}$ at $\frac{P_{\mathrm{PIN2}}^{\mathrm{right}}}{P_{\mathrm{PIN2}}^{\mathrm{left}}} = 1$, permeabilities as in the main model $P_{\mathrm{AUX1}}^{0}, P_{\mathrm{PIN2}}^{0\ \mathrm{initial}}$. Right: steady-state relative $[\mathrm{IAA}]_{\mathrm{cell}}$ at PIN2 asymmetry with higher PIN2 in the left column ($\frac{P_{\mathrm{PIN2}}^{\mathrm{right}}}{P_{\mathrm{PIN2}}^{\mathrm{left}}} = 0.5$); AUX1 permeability unchanged $\mathrm{P}_{\mathrm{AUX1}}^{0}$. **d)** $\frac{\mathrm{IAA}^{\mathrm{left}}}{\mathrm{IAA}^{\mathrm{right}}}(k)$ for (a,b,c) models, our main model and realistic geometry model for "no stimulation" cases. $\frac{\mathrm{IAA}^{\mathrm{left}}}{\mathrm{IAA}^{\mathrm{right}}}$ is proportional to $\frac{P_{\mathrm{PIN2}}^{\mathrm{upper}}}{P_{\mathrm{PIN2}}^{\mathrm{lower}}} = k$ for all models tested. Color bar for all panels: relative concentration $[\mathrm{IAA}]_{\mathrm{cell}}/[\mathrm{IAA}]_{\mathrm{shoot}}$.

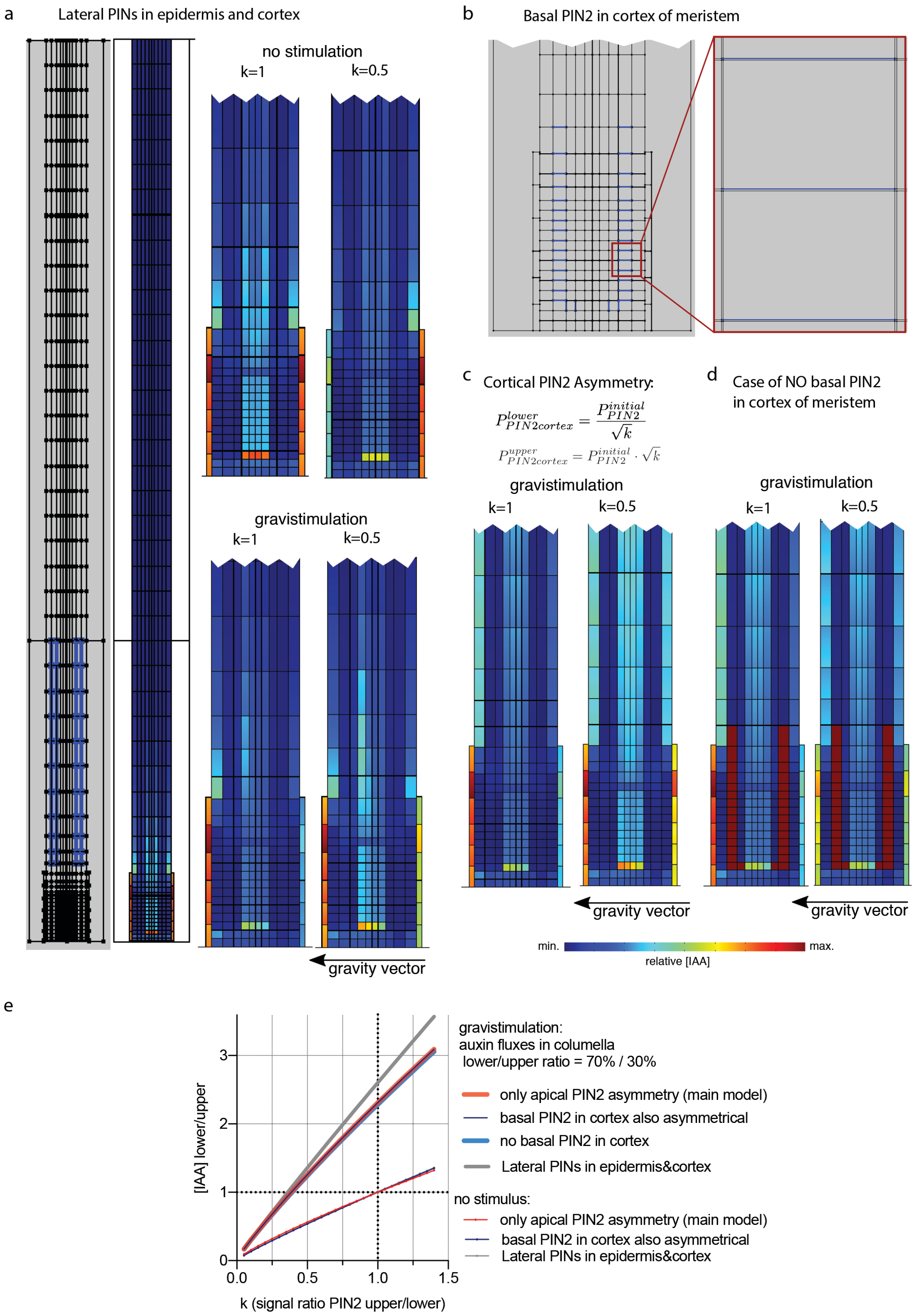

Supplementary Figure 14. Effect of PIN2 localization on auxin distribution. **a)** Effect of lateral PIN2 localization in epidermis and cortex cells. Left: scheme of additional lateral PIN2 localization in elongated geometry (blue lines highlighting inner lateral membranes of epidermis and cortex); other permeabilities are the same as in the main model; steady-state

relative $[IAA]_{cell}$ at $P_{PIN2}^{lateral} = P_{PIN2}^{0\ initial}$ and $\frac{P_{PIN2cortical}^{upper}}{P_{PIN2cortical}^{lower}} = k = 1$. Right, upper panel: zoom of the root apex, steady-state relative $[IAA]_{cell}$ at $k = 1$ and $k = 0.5$ for "no stimulation" case. Right, lower panel: steady state relative $[IAA]_{cell}$ at $k = 1$ and $k = 0.5$ for gravistimulation strength $\frac{70\%}{30\%}$. **b)** Scheme of basal PIN2 in cortex cells of the root meristem (zoom of the root apex), blue lines highlighting lower membranes of cortical cells. **c)** Steady-state relative $[IAA]_{cell}$ at $k = 1$ and for PIN2 asymmetry present both in apical PIN2 and basal cortical PIN2 ($k = 0.5,$), for gravistimulation strength $\frac{70\%}{30\%}$. **d)** Case of no PIN2 on the basal side of cortical cells ($P_{PIN2cortical}^{basal} = 0$). Steady-state relative $[IAA]_{cell}$ at $k = 1$ and $k = 0.5$ for gravistimulation strength $\frac{70\%}{30\%}$. This leads to $[IAA]_{cell}$ accumulation in cortex cells, but has no effect on $\frac{IAA^{lower}}{IAA^{upper}}(k)$. **e)** $\frac{IAA^{lower}}{IAA^{upper}}(k)$ of the models tested (a,c,d) under "no stimulation" and "gravistimulation" conditions, compared to the main model.

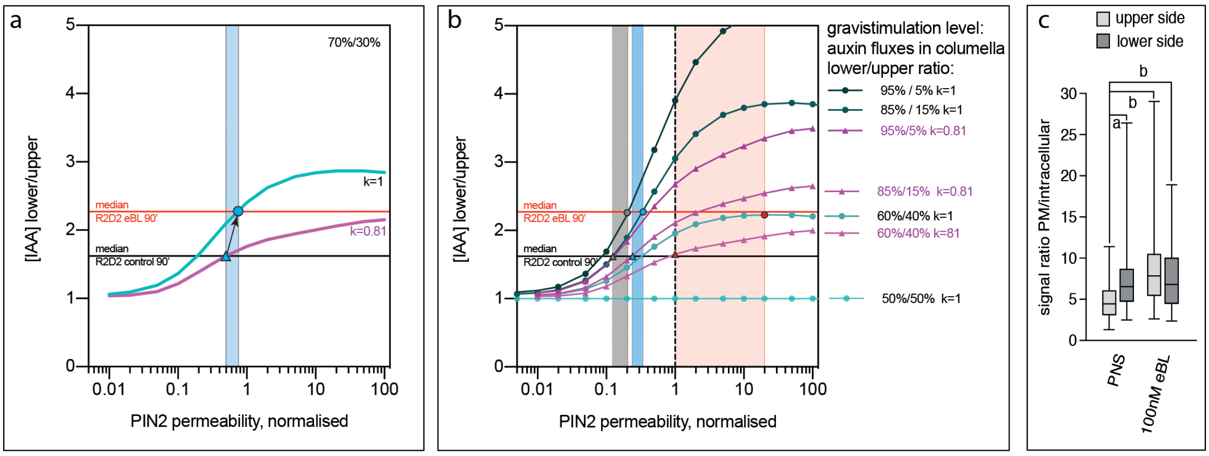

Supplementary Figure 15. Effect of $P_{\mathrm{PIN2}}^{\mathrm{initial}}$ values on $\frac{\mathrm{IAA}^{\mathrm{lower}}}{\mathrm{IAA}^{\mathrm{upper}}}$ in presence of PIN2 asymmetry. **a)** Dependence of $\frac{\mathrm{IAA}^{\mathrm{lower}}}{\mathrm{IAA}^{\mathrm{upper}}}$ values on initial PIN2 permeability in gravistimulated roots, for $k = 1$ and $k = 0.81$, and identical gravistimulation strength in the root tip. The shaded blue area marks the PIN2 permeability increase required for shifting auxin ratios from control values (blue triangle) to eBL-treated values (blue circle). **b)** The same graph as in (a), but simulating different gravistimulation strengths, illustrates the required changes in PIN2 permeability (shaded bands). **c)** PIN2:Venus signals at the PM of root epidermis cells in the root EZ, grown on control medium (PNS) or pre-treated with 100 nM eBL for 16 hours, after gravistimulation for 150 minutes. Signal intensities were obtained by determination of grey values at the PM, followed by normalization to corresponding intracellular grey values. 36-45 cells in 12-15 roots were analyzed for each dataset. One-way ANOVA with post-hoc Tukey HSD test was used to determine p-values (‘a’: p<0.05; ‘b’: p<0.01). Whiskers in the box plots represent the entire range of outliers obtained in the datasets; grey boxes display first and third quartiles; center line: median. Source data are provided as Source Data files.

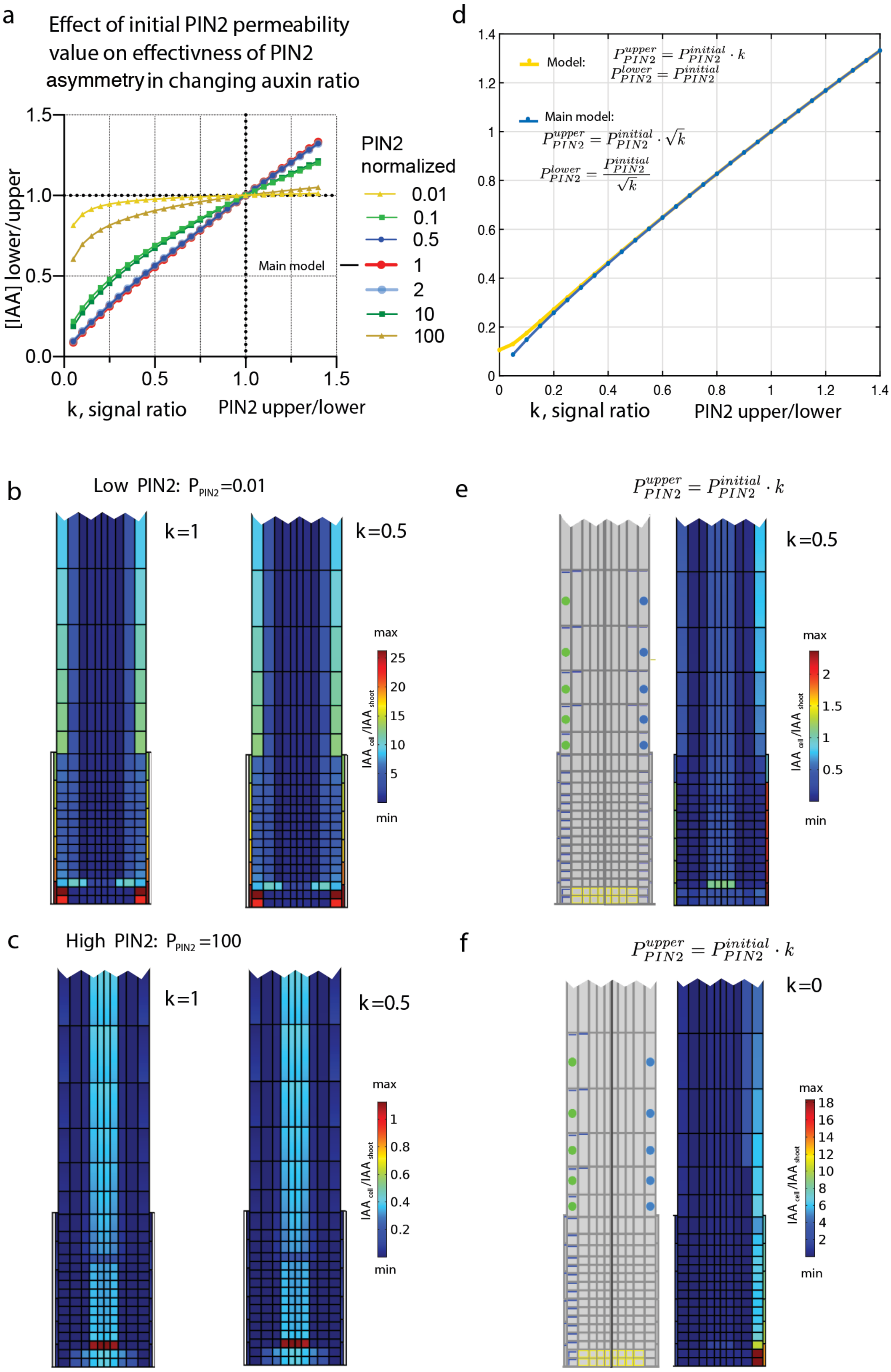

Supplementary Figure 16. Effect of $P_{\mathrm{PIN2}}^{\mathrm{initial}}$ values on the slope of the $\frac{\mathrm{IAA}^{\mathrm{lower}}}{\mathrm{IAA}^{\mathrm{upper}}}(k)$ curve. **a)** Modelling results for different initial levels of PIN2 $P_{\mathrm{PIN2}}^{\mathrm{initial}}$, with PIN1 and AUX1 permeabilities remaining constant. $\frac{\mathrm{IAA}^{\mathrm{lower}}}{\mathrm{IAA}^{\mathrm{upper}}}$ is calculated by dividing an average $[\mathrm{IAA}]_{\mathrm{cell}}$ in the first 5 cells of

the elongation zone on the lower side (marked by green circles in (e) and in Fig. 8a) and on the upper side (marked by blue circles). For very low and very high levels of PIN2, $\bar{P}_{\mathrm{PIN2}}^{\mathrm{initial}} \gg 1$ and $\bar{P}_{\mathrm{PIN2}}^{\mathrm{initial}} \ll 1$, changes in auxin concentration due to PIN2 asymmetry are very subtle ($\frac{\mathrm{IAA}^{\mathrm{lower}}}{\mathrm{IAA}^{\mathrm{upper}}}$~=1 for any k). PIN2 permeability is normalized by $P_{\mathrm{PIN2}}^{0\ \mathrm{initial}}$. **b)** Auxin distribution in case when PIN2 permeability is low: $P_{\mathrm{PIN2}}^{\mathrm{initial}} = 0.01$. Left panel, $k = 1$: Auxin accumulates in epidermal cells of the elongation zone due to AUX1 activity prevailing over PIN2. Right panel, $k = 0.5$: PIN2 asymmetry does not result in IAA asymmetry. **c)** Auxin distribution in case when PIN2 permeability is high: $P_{\mathrm{PIN2}}^{\mathrm{initial}} = 100$. Left panel, $k = 1$: Auxin concentration in epidermal cells of elongation zone is low due to prevailing PIN2 activity over AUX1. Right panel, $k = 0.5$: PIN2 asymmetry does not result in IAA asymmetry. **d)** Comparison of results of the main model (where both, upper PIN2 permeability and lower PIN2 permeability are changed reciprocally; blue line) and of a simulation, in which only PIN2 permeability at the upper side decreases during onset of the PIN2 asymmetry (yellow line). $\frac{\mathrm{IAA}^{\mathrm{lower}}}{\mathrm{IAA}^{\mathrm{upper}}}(k)$ curves coincide, indicating, that the ratio of PIN2 permeabilities ($\frac{P_{\mathrm{PIN2}}^{\mathrm{upper\ side}}}{P_{\mathrm{PIN2}}^{\mathrm{lower\ side}}}$), rather than absolute values of PIN2 permeabilities, is important for establishing $\frac{\mathrm{IAA}^{\mathrm{lower}}}{\mathrm{IAA}^{\mathrm{upper}}}$ asymmetry. **e)** IAA distribution at $k = 0.5$ is the same for both setups introduced in (d) (also, compare to Fig. 8b). **f)** IAA distribution for $k = 0$ (PIN2 permeability on the upper side is zero) shows high accumulation of auxin in the meristem. Independently of this accumulation, $\mathrm{IAA}^{\mathrm{upper}}$ is increased and $\frac{\mathrm{IAA}^{\mathrm{lower}}}{\mathrm{IAA}^{\mathrm{upper}}} < 1$.

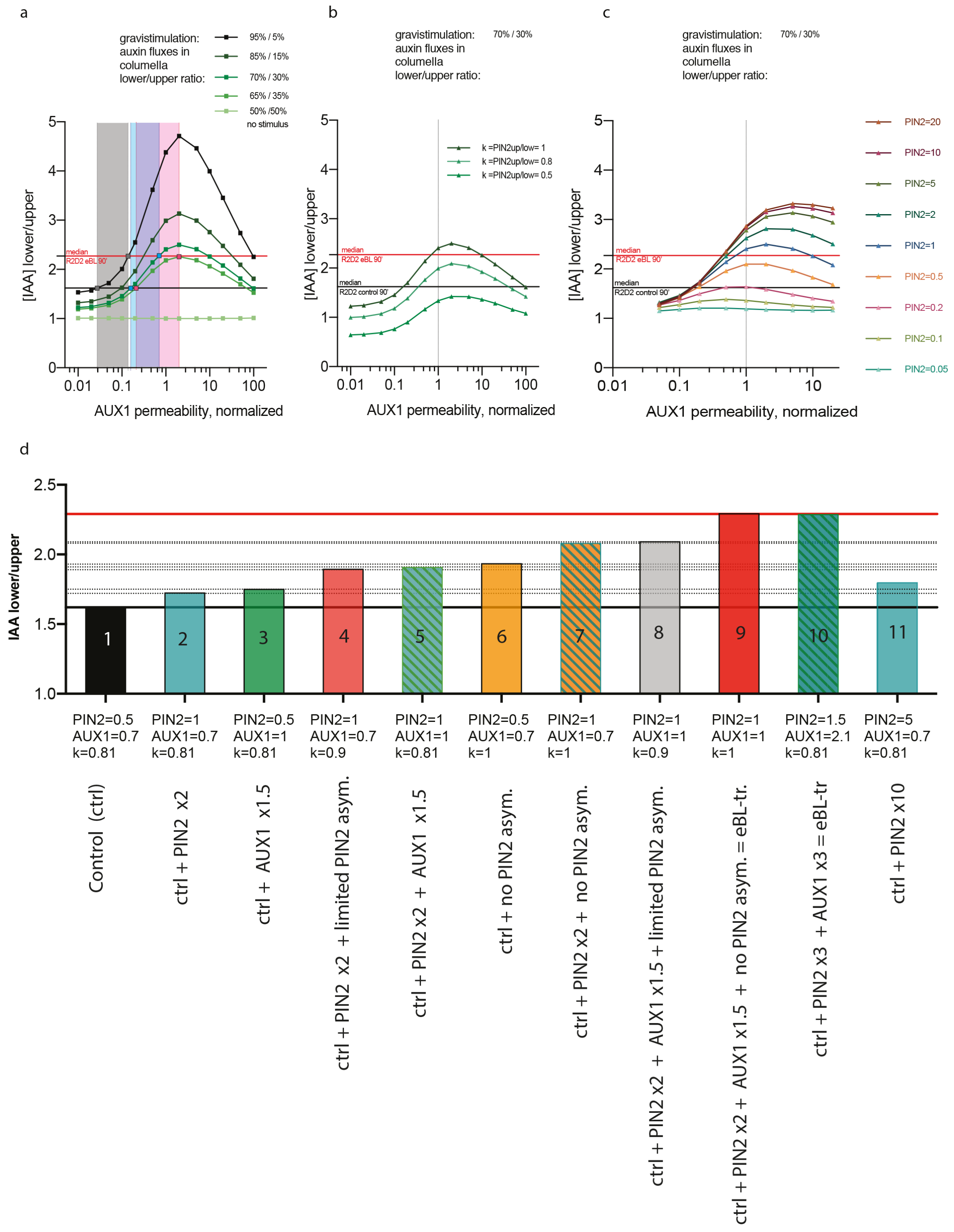

Supplementary Figure 17. Effect of AUX1, PIN2 permeability and gravistimulation strength and their combination on auxin ratio. **a)** $\frac{\mathrm{IAA}^{\mathrm{lower}}}{\mathrm{IAA}^{\mathrm{upper}}}(P_{\mathrm{AUX1}}/P^{0}_{\mathrm{AUX1}})$ for the range of different gravistimulation strengths, indicated. $P^{\mathrm{initial}}_{\mathrm{PIN2}} = P^{0\,\mathrm{initial}}_{\mathrm{PIN2}}$. Grey shading: minimal shift in AUX1 permeability required, to increase $\frac{\mathrm{IAA}^{\mathrm{lower}}}{\mathrm{IAA}^{\mathrm{upper}}}$ from 1.62 to 2.3 (grey dots) under conditions of high gravistimulation strength (auxin flux ratio 95%/5%). Blue shading: shift in

AUX1 permeability required for increasing $\frac{\mathrm{IAA}^{\mathrm{lower}}}{\mathrm{IAA}^{\mathrm{upper}}}$ from 1.62 to 2.3 $(\mathrm{blue\ dots})$ at an auxin flux ratio of 70%/30%. Pink shading: shift in AUX1 permeability required for increasing $\frac{\mathrm{IAA}^{\mathrm{lower}}}{\mathrm{IAA}^{\mathrm{upper}}}$ from 1.62 to 2.3 (pink dots) at auxin flux ratio 65%/35%. log scale. **b)** $\frac{\mathrm{IAA}^{\mathrm{lower}}}{\mathrm{IAA}^{\mathrm{upper}}}\left(P_{\mathrm{AUX1}}/P^{0}_{\mathrm{AUX1}}\right)$ for different levels of PIN2 asymmetry $\frac{P^{\mathrm{upper\ side}}_{\mathrm{PIN2}}}{P^{\mathrm{lower\ side}}_{\mathrm{PIN2}}} = k$. $P^{\mathrm{initial}}_{\mathrm{PIN2}} = P^{0\ \mathrm{initial}}_{\mathrm{PIN2}}$, gravistimulation strength $\frac{70\%}{30\%}$; log scale. **c)** $\frac{\mathrm{IAA}^{\mathrm{lower}}}{\mathrm{IAA}^{\mathrm{upper}}}\left(P_{\mathrm{AUX1}}/P^{0}_{\mathrm{AUX1}}\right)$ for the range of different $P^{\mathrm{initial}}_{\mathrm{PIN2}}$; $k = 1$, gravistimulation strength $\frac{70\%}{30\%}$; log scale. **d)** Comparison of $\frac{\mathrm{IAA}^{\mathrm{lower}}}{\mathrm{IAA}^{\mathrm{upper}}}$ values for an exemplified simulation with control root conditions as follows: $\bar{P}^{\mathrm{initial}}_{\mathrm{PIN2}} = 0.5$, $\bar{P}_{\mathrm{AUX1}} = 0.7$, $k = 0.81$ (bar #1). In further simulations $\bar{P}^{\mathrm{initial}}_{\mathrm{PIN2}}$, $\bar{P}_{\mathrm{AUX1}}$ and PIN2 asymmetry (k) have been modified. These simulations demonstrate the relative contributions of individual parameters as well as combinatorial effects of parameters (bars #2-8) to the increase of $\frac{\mathrm{IAA}^{\mathrm{lower}}}{\mathrm{IAA}^{\mathrm{upper}}}$. Note that the effect of PIN2 asymmetry (k=0.81; bar #1 vs k=1; bar #6) is higher than effects of a twofold increase in $\bar{P}^{\mathrm{initial}}_{\mathrm{PIN2}}$ (bar #2) or $\bar{P}_{\mathrm{AUX1}}$ (bar #3) alone. Bar #10 represents $\frac{\mathrm{IAA}^{\mathrm{lower}}}{\mathrm{IAA}^{\mathrm{upper}}}$ values in eBL-treated roots achieved by elimination of PIN2 asymmetry, together with a mild increase in $\bar{P}^{\mathrm{initial}}_{\mathrm{PIN2}}$ and $\bar{P}_{\mathrm{AUX1}}$. Bar #11 shows that $\frac{\mathrm{IAA}^{\mathrm{lower}}}{\mathrm{IAA}^{\mathrm{upper}}}$ in eBL-treated roots requires a simultaneous threefold increase in $\bar{P}^{\mathrm{initial}}_{\mathrm{PIN2}}$ and $\bar{P}_{\mathrm{AUX1}}$, provided that PIN2 asymmetry is unchanged, when compared to the control (bar #1).

SUPPLEMENTARY REFERENCES

1. Fendrych M, *et al.* Rapid and reversible root growth inhibition by TIR1 auxin signalling. *Nat Plants* **4**, 453-459 (2018).

2. Swarup R, *et al.* Root gravitropism requires lateral root cap and epidermal cells for transport and response to a mobile auxin signal. *Nat Cell Biol* **7**, 1057-1065 (2005).

3. Band LR, *et al.* Systems analysis of auxin transport in the Arabidopsis root apex. *Plant Cell* **26**, 862-875 (2014).

4. Grieneisen VA, Xu J, Maree AF, Hogeweg P, Scheres B. Auxin transport is sufficient to generate a maximum and gradient guiding root growth. *Nature* **449**, 1008-1013 (2007).

5. van den Berg T, Korver RA, Testerink C, Ten Tusscher KH. Modeling halotropism: a key role for root tip architecture and reflux loop remodeling in redistributing auxin. *Development* **143**, 3350-3362 (2016).

6. Kramer EM, Frazer NL, Baskin TI. Measurement of diffusion within the cell wall in living roots of Arabidopsis thaliana. *J Exp Bot* **58**, 3005-3015 (2007).

7. Rutschow HL, Baskin TI, Kramer EM. The carrier AUXIN RESISTANT (AUX1) dominates auxin flux into Arabidopsis protoplasts. *New Phytol* **204**, 536-544 (2014).

8. Delbarre A, Muller P, Imhoff V, Guern J. Comparison of mechanisms controlling uptake and accumulation of 2,4-dichlorophenoxy acetic acid, naphthalene-1-acetic acid, and indole-3-acetic acid in suspension-cultured tobacco cells. *Planta* **198**, 532-541 (1996).

9. Carrier DJ, *et al.* The binding of auxin to the Arabidopsis auxin influx transporter AUX1. *Plant Physiol* **148**, 529-535 (2008).

10. Kramer EM, Rutschow HL, Mabie SS. AuxV: a database of auxin transport velocities. *Trends Plant Sci* **16**, 461-463 (2011).

11. Kramer EM, Ackelsberg EM. Auxin metabolism rates and implications for plant development. *Front Plant Sci* **6**, 150 (2015).

12. Band LR*, et al.* Root gravitropism is regulated by a transient lateral auxin gradient controlled by a tipping-point mechanism. *Proc Natl Acad Sci U S A* **109**, 4668-4673 (2012).

13. Jonas AR, *et al.* Auxin transport through non-hair cells sustains root-hair development. *Nat Cell Biol.* **11**, 78–84 (2009).
